# Novel physical performance-based models for activities of daily living disability prediction among Chinese older community population: a nationally representative survey in China

**DOI:** 10.1186/s12877-022-02905-y

**Published:** 2022-03-31

**Authors:** Li Zhang, Yueqiao Chen, Jing Liu, Yifan Yu, Huijie Cui, Qiuzhi Chen, Kejin Chen, Chunxia Yang, Yanfang Yang

**Affiliations:** Department of Epidemiology and Biostatistics, West China School of Public Health and West China Fourth Hospital, Sichuan University, No.17 Section 3, Renmin South Road, Chengdu, 610041 Sichuan China

**Keywords:** Prediction model, Physical performance, Activities of daily living disability, Short physical performance battery, Gait speed

## Abstract

**Background:**

Physical performances including upper and lower limb functions have predictive roles in activities of daily living (ADL) disability, but they have rarely been incorporated into prediction models. This study primarily aimed to develop and validate novel physical performance-based models for ADL disability among Chinese older adults. Comparisons of predictive performance across multiple models were performed, and model simplification was further explored.

**Methods:**

Data were obtained from the China Health and Retirement Longitudinal Study in the 2011 and 2015 waves, containing 2192 older adults over 60 years old. Our models were constructed by logistic regression analysis, using a backward stepwise selection. Model performance was internally validated by discrimination, calibration, and clinical utility. Integrated Discrimination Improvement (IDI) and Net Reclassification Improvement (NRI) were used to assess the incremental benefit of the extended models. Moreover, nomograms were built for visualization.

**Results:**

We selected gender, age, smoking, self-report health condition, BMI, depressive symptoms, and cognitive function into the fundamental model (Model 1). Based on Model 1, five novel prediction models were constructed by adding handgrip strength (Model 2), Short Physical Performance Battery (SPPB) (Model 3), gait speed (Model 4), handgrip strength plus SPPB (Model 5), and handgrip strength plus gait speed (Model 6), respectively. Significant improvement in predictive values were observed for all five novel models compared with Model 1 (C-index = 0.693). The lower limb model (Model 3 SPPB model: C-index = 0.731) may play a key role in the prediction of ADL disability, reflecting a comparable predictive value to the comprehensive models combining both upper and lower limbs (Model 5 handgrip strength + SPPB model: C-index = 0.732). When we simplified the lower limb models by replacing SPPB with gait speed, the predictive values attenuated slightly (C-index: Model 3 vs Model 4: 0.731 vs 0.714; Model 5 vs Model 6: 0.732 vs 0.718), but still better than the upper limb model (Model 2 handgrip strength model: C-index = 0.701).

**Conclusions:**

Physical performance-based models, especially lower limb model, provided improved prediction for ADL disability among Chinese older adults, which may help guide the targeted intervention.

**Supplementary Information:**

The online version contains supplementary material available at 10.1186/s12877-022-02905-y.

## Background

Activities of daily living (ADL) disability, defined as a dependency in performing normal daily activities, is a significant and consequential health indicator of the older population [1]. A growing number of older adults, as their ages advance, lose independence in performing ADL, leading to multiple adverse events such as falls, hospitalization, and mortality [2–4]. Predicting and identifying high-risk individuals for ADL disability is a core goal of health aging management. Predictors including demographic characteristics, chronic conditions, and health behaviors for ADL disability have been well described [5–8], but multi-factorial prediction models have not been adequately explored, with limited factors or lack of validation [9–12]. Therefore, ADL disability prediction tools for the older community population remain an unmet need.

Physical performances including upper limb index (eg. handgrip strength) [13] and lower limb index (eg. gait speed and the short physical performance battery (SPPB)) [14] have proved to be crucial factors of intrinsic capacity in older adults [15]. Substantial evidence shows that physical performances are strong and independent predictors of ADL disability, with satisfactory validity in the older population [16–19]. Despite that, these predictors have rarely been incorporated in most ADL disability prediction tools, with very few studies reporting on the prediction models with physical performance [9, 10, 12, 20]. Among these limited studies, most of them lacked adequate validation for use in clinical practice [9, 10, 12] or limited by a small sample size [20] and long follow-up interval [20]. Thus, it is necessary to further understand the added value of physical performance on ADL disability risk prediction models and to refine the prediction tools among the older community population. In addition, these physical performance-based models can be categorized into upper limb model, lower limb model, and comprehensive model combining both. However, it was still unclear which model has the best predictive value. In particular, gait speed, a crucial component of SPPB, is regarded as an objective and reliable tool for predicting ADL disability. Accumulating studies have indicated that assessing gait speed alone performed as well as the full SPPB tests for the prediction of disability [10, 14]. Therefore, whether the full SPPB model could be simplified to the gait speed model is worth exploring.

Hence, this study primarily aimed to derive prediction models that incorporated traditional risk factors and physical performances for the prediction of ADL disability among Chinese older adults, while also determining if the physical performance-based models improve the predictive value compared to the fundamental model. Secondly, we performed comparisons across different physical performance-based models to determine the optimal one. Finally, we also explored the possibility of full SPPB model simplified to gait speed model.

## Methods

All methods were performed in accordance with the relevant guidelines and regulations.

### Study design and participants

Data were obtained from China Health and Retirement Longitudinal Study (CHARLS), a nationally representative longitudinal survey conducted by Peking University among Chinese middle-aged and older adults. The CHARLS baseline survey was conducted from 2011 to 2012, covering 150 counties in 28 provinces of China. A wide range of information on socioeconomic status, health circumstances, as well as anthropometric and laboratory measurements, were collected [21]. The participants were followed in 2013, 2015, and 2018 through face-to-face computer-assisted personal interview (CAPI), respectively. Detailed descriptions of the survey design and procedures were available elsewhere [21].

In this study, we restricted our analysis to a subset of participants aged 60 years and older, without ADL disability at the baseline survey of CHARLS (2011 wave). At baseline, a total of 2840 participants with missing information on key variables such as all physical performances and ADL status were excluded, and 4303 well-functioning participants were included for analyses. Compared with the excluded participants, the included participants were older and more likely to be females, with worse demographic characteristics, chronic conditions, and health behaviors (Table S1). After a 4-year follow-up, 2111 were lost to follow-up and 2192 participants reported complete information on the ADL outcome, and both groups shared similar baseline characteristics (Fig. S1 and Table S2).

All the participants signed informed consent at the time of participation and this study was approved by the Institutional Review Board of Peking University (IRB00001052–11014).

### Outcome

ADL was evaluated by the Katz ADL scale referring to daily self-care tasks, including taking a bath, eating, getting in and out of bed, dressing, using the toilet, and maintaining continence of urine and feces [22]. In this study, participants were determined as having ADL disability if they reported needing any help in at least one of these ADL items [23].

### Physical performances—handgrip strength

We assessed the upper limb function by performing the handgrip strength test. Subjects were asked to stand and hold the dynamometer at a right angle (90°), squeezing the handle as hard as possible for a few seconds. Each hand was measured twice in turn. In this study, the maximum handgrip strength (kg) from all four attempts was used to measure handgrip strength [24].

### Physical performances— the short physical performance battery (SPPB)

We evaluated the lower limb function by conducting the SPPB, which includes three measurements of balance, gait speed, and repeated chair stands tests. In the balance test, participants were asked to take two of the following balance tests: side-by-side stand, semi-tandem stand, and full tandem stand. All participants were asked to conduct a semi-tandem stand. If participants were able to hold a semi-tandem stand for 10s, they were then asked to perform the full tandem stand for 30s (for participants aged 70 or above) or 60s (for participants aged less than 70). Otherwise, they were asked to conduct a side-by-side stand for about 10s [25]. In the gait speed test, subjects walked twice (there and back) along a 2.5-m straight road at their usual speed and the time taken was recorded [25–28]. For repeated chair stands test, subjects were asked to stand and sit in a chair five times as quickly as possible with their arms crossed over their chest. The time was measured from the moment the subjects started to stand up until they were fully standing after rising for the fifth time [25]. Each test was scored from 0 to 4. The balance test score depended on the hierarchical combination of performance on the three kinds of balance tests. In the other two tests, score 0 was assigned to those who were unable to complete the tests, and scores from 1 to 4 were assigned according to the quartiles of time required to complete the tests [29]. Additionally, the SPPB score was obtained by summing balance, gait speed, and repeated chair stands tests, ranging from 0 (worst performance) and 12 (best performance).

### Physical performances—gait speed

Gait speed was one part of the SPPB and has been given detailed descriptions in the SPPB section. The average speed of the two trials was used in the analysis [25].

### Other predictors

The following variables were also considered as predictors: age, gender, marital status, education, social activity, drinking, smoking, night sleep, comorbidities, body mass index (BMI), self-assessment of health conditions, depressive symptoms, and cognitive function [30, 31]. Age was classified into the following four groups: 60–64, 65–69, 70–74, and older than 75 years old [32]. Marital status was categorized into married or cohabiting, widowed, and another marital status including separated, divorced, and never married. Education was categorized into the following five categories: illiterate, primary school, middle school, high school, and college and above [32]. Social activity frequencies were classified as never, not regularly, almost weekly, and almost daily [28]. Drinking was divided into the following four categories: never, quit drinking, less than once a month, and more than once a month [28]. Smoking was classified into the following four categories, never, quit smoking, less than 20 cigarettes a day, and more than 20 cigarettes a day [28]. Moreover, night sleep durations were classified as less than 6 h, 6 to 9 h, and more than 9 h [28]. Suffering from two or more self-report chronic diseases was defined as comorbidity condition [28]. BMI was classified according to WHO cut-off points for Chinese: underweight (BMI < 18.5 kg/m^2^), normal weight (BMI = 18.5 kg/m^2^ to 23.9 kg/m^2^), overweight (BMI = 24 kg/m^2^ to 27.9 kg/m^2^) and obese (BMI ≥ 28 kg/m^2^) [33]. Self-report health condition was classified into good, fair, poor, and very poor. Cognitive function was assessed by two domains, episodic memory and mental intactness, with global cognitive scores ranging from 0 to 21 [25, 34]. The episodic memory score was defined as the average of the immediate and delayed recall scores, with the scores ranging from 0 to 10 [34]. In CHARLS, the mental intactness tests included serial subtraction of 7 from 100 (up to five times), the date (month, day, and year), the day of the week, the season of the year, and intersecting pentagon copying test. Answers to these questions were summed into a mental intactness score ranging from 0 to 11 [34]. Depressive symptoms were measured using Center for Epidemiologic Studies Depression Scale-10 items (CES-D-10) (ranging from 0 to 30). Participants with scores ≥10 were considered to have significant depressive symptoms [35].

### Statistical analysis

A descriptive analysis was performed to characterize the study populations. Continuous variables were reported as median and quartile (non-normal distribution), and categorical variables were reported as numbers and percentages. We compared the baseline characteristics between ADL status using the Kruskal-Wallis test for continuous variables or the chi-square test for categorical variables.

We established six logistic regression models using logistic regression analysis. Model 1 (fundamental model) was established using a backward stepwise selection with the Akaike information criterion (AIC). We selected seven predictors (gender, age, smoking, self-report health condition, BMI, depressive symptoms, and cognitive function) from 13 candidate predictors (age, gender, marital status, education, social activity, drinking, smoking, night sleep, comorbidity, self-report health condition, BMI, depressive symptoms, and cognitive function). Besides, five physical performance-based models were established based on Model 1, adding handgrip strength (Model 2), SPPB (Model 3), gait speed (Model 4), handgrip strength plus SPPB (Model 5), and handgrip strength plus gait speed (Model 6), respectively. In our study, Model 2 represented upper limb model, Model 3 to 4 represented lower limb model, and Models 5 to 6 severed as comprehensive model combining both upper and lower limbs. Predictors selected through every model were considered of odds ratio (OR) and corresponding 95% confidential interval (CI). Moreover, we transformed each model into visualized nomogram, facilitating risk probability calculation using more concrete numbers for individuals.

The model performance was evaluated by discrimination, calibration, and clinical utility. The discrimination was quantified by the concordance index (C-index) which was equivalent to the area under the receiver-operating characteristic curve (AUC) in a logistic analysis. The AUC closer to 1 represented better discriminant ability, and AUC closer to 0.5 the opposite [36]. C-index ≥0.70 defined good discrimination [37]. We used the calibration plots to assess the calibration of the model by comparing the consistency between the actual outcomes and predicted outcomes. The 45-degree line represented perfect calibration, and adjacency to this line indicated good calibration [38]. Clinical decision curve analysis (DCA) was conducted to determine the clinical utility of the model by quantifying the net benefits at threshold probability [39]. Interventions would be made only when the outcome probability reached the threshold value. Moreover, we validated our models internally by conducting 1000 bootstrap resamples to generate the bootstrap-corrected C-index and calibration plots.

We also used Integrated Discrimination Improvement (IDI) and Net Reclassification Improvement (NRI) to assess the incremental benefit in the subsequent extended models (Model 2 to Model 6). The IDI index shows the average net improvement in the predicted risk for ADL disability in the extended models [40, 41]. The NRI index can be interpreted as the proportion of correct risk reclassification after adding physical performances to Model 1 [40]. Category-free NRI was adopted due to the lack of consensus on categorization of ADL disability risk in the older community population. In general, NRI (IDI) > 0 is considered relatively positive incremental benefit in the subsequent new models, indicating better prediction performance than the old one.

All statistical analyses were performed with the use of R software (version 3.0.2; http://www.Rproject.org) and SPSS (version 20.0). All statistical tests were two-sided, and significance was set as *P* value<0.05.

## Results

### Characteristics of participants

During the 4-year follow-up, 2192 participants reported the ADL outcome, with 311 (14.2%) reporting ADL disability. Comparisons of baseline characteristics between older adults with ADL disability and those without ADL disability at follow-up were displayed in Table 1. Most variables were associated with ADL status except marital status, social activity, drinking, and comorbidity (*P* ≥ 0.05). Compared with the participants without ADL disability, those who developed ADL disability tended to have lower SPPB score (7.00 vs 8.00, *P* < 0.001), weaker handgrip strength (22.00 kg vs 25.32 kg, *P* < 0.001), and lower gait speed (0.51 m/s vs 0.61 m/s, *P* < 0.001) at baseline.

**Table 1:** Baseline characteristics in baseline and 2015 follow-up cohort

	N (%) or Median(Q1-Q3)			
**Variables**	Baseline	4-year follow-up		
	All samples	ADL disability	ADL independent	*P* value
**Overall**	2192	311	1881	
**Age (Years)**				< 0.001^*^
60 ~	809 (36.9%)	82 (26.4%)	727 (38.6%)	
65 ~	569 (26.0%)	63 (20.3%)	506 (26.9%)	
70 ~ −	423 (19.3%)	75 (24.1%)	348 (18.5%)	
75 ~	391 (17.8%)	91 (29.3%)	300 (15.9%)	
**Gender**				0.045^*^
Male	892 (40.7%)	110 (35.4%)	782 (41.6%)	
Female	1300 (59.3%)	201 (64.6%)	1099 (58.4%)	
**Marital status**				0.551
Married/Cohabitated	1680 (76.6%)	233 (74.9%)	1447 (76.9%)	
Widowed	468 (21.4%)	73 (23.5%)	395 (21.0%)	
Other	44 (2.01%)	5 (1.61%)	39 (2.07%)	
**Education**				0.043^*^
Illiterate	911 (41.6%)	153 (49.2%)	758 (40.3%)	
Primary school	973 (44.4%)	126 (40.5%)	847 (45.0%)	
Middle school	214 (9.76%)	24 (7.72%)	190 (10.1%)	
High school	70 (3.19%)	6 (1.93%)	64 (3.40%)	
College and above	24 (1.09%)	2 (0.64%)	22 (1.17%)	
**Social activity**				0.327
Never	1163 (53.1%)	155 (49.8%)	1008 (53.6%)	
Not regularly	261 (11.9%)	36 (11.6%)	225 (12.0%)	
Almost Weekly	212 (9.67%)	28 (9.00%)	184 (9.78%)	
Almost daily	556 (25.4%)	92 (29.6%)	464 (24.7%)	
**Smoking**				0.026^*^
Never	1482 (67.6%)	214 (68.8%)	1268 (67.4%)	
Quit	268 (12.2%)	40 (12.9%)	228 (12.1%)	
Less than 20 /day	212 (9.67%)	38 (12.2%)	174 (9.25%)	
More than 20 /day	230 (10.5%)	19 (6.11%)	211 (11.2%)	
**Drinking**				0.199
Never	1422 (64.9%)	207 (66.6%)	1215 (64.6%)	
Quit	237 (10.8%)	41 (13.2%)	196 (10.4%)	
Less than once/month	116 (5.29%)	15 (4.82%)	101 (5.37%)	
More than once/month	417 (19.0%)	48 (15.4%)	369 (19.6%)	
**Night sleep (hours)**				0.021*
6–9	1216 (55.5%)	150 (48.2%)	1066 (56.7%)	
< 6	807 (36.8%)	132 (42.4%)	675 (35.9%)	
> =9	169 (7.71%)	29 (9.32%)	140 (7.44%)	
**Comorbidity**				0.425
0	471 (21.5%)	63 (20.3%)	408 (21.7%)	
1	633 (28.9%)	83 (26.7%)	550 (29.2%)	
≥ 2	1088 (49.6%)	165 (53.1%)	923 (49.1%)	
**BMI**				< 0.001 ^*^
Normal	1140 (52.0%)	141 (45.3%)	999 (53.1%)	
Underweight	201 (9.2%)	34 (10.9%)	167 (8.9%)	
Overweight	613 (28.0%)	76 (24.4%)	537 (28.5%)	
Obese	238 (10.9%)	60 (19.3%)	178 (9.46%)	
**Self-report health**				< 0.001^*^
Good	200 (9.12%)	23 (7.40%)	177 (9.41%)	
Fair	616 (28.1%)	71 (22.8%)	545 (29.0%)	
Poor	877 (40.0%)	116 (37.3%)	761 (40.5%)	
Very poor	499 (22.8%)	101 (32.5%)	398 (21.2%)	
**Depression symptoms**				< 0.001^*^
Normal	1180 (53.8%)	132 (42.4%)	1048 (55.7%)	
Depression	1012 (46.2%)	179 (57.6%)	833 (44.3%)	
**Cognitive function**	9.00 [5.50;12.50]	7.00 [4.00;10.50]	9.50 [6.00;12.50]	< 0.001^*^
**Handgrip strength (kg)**	24.75 [19.48;31.50]	22.00 [16.88;27.75]	25.32 [20.00;32.00]	< 0.001^*^
**Gait speed (m/s)**	0.59 [0.45;0.73]	0.51 [0.37;0.64]	0.61 [0.46;0.75]	< 0.001^*^
**SPPB score**	8.00 [6.00;10.0]	7.00 [5.00;9.00]	8.00 [7.00;10.0]	< 0.001^*^

*Note*. *ADLs* activities of daily living, *BMI* body mass index, *SPPB* Short Physical Performance Battery

*p < 0.05

### Development and internal validation of models

**Fig. 1:**
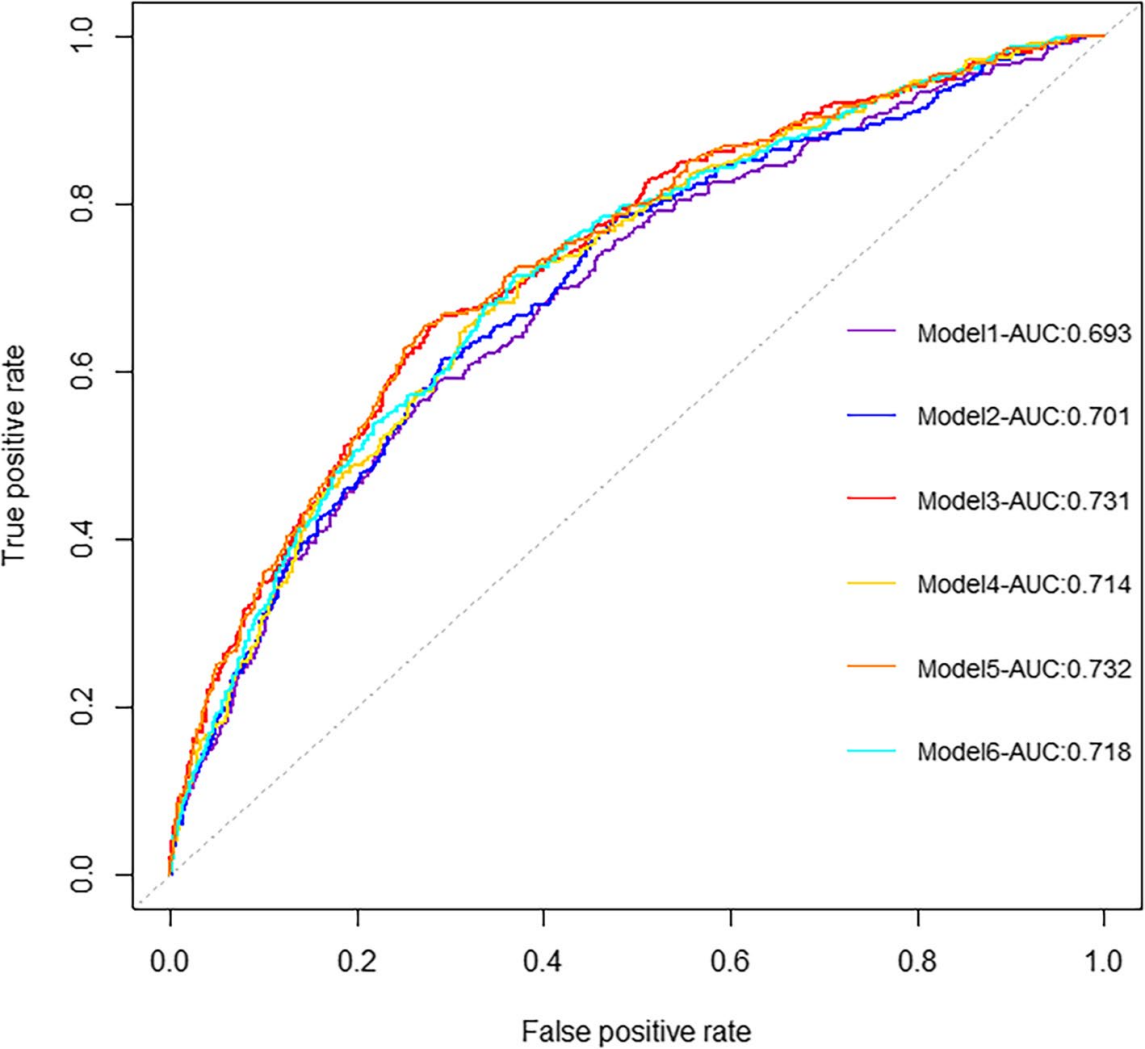
The ROC curves for different prediction models. Note. AUC = area under the receiver-operating characteristic curve; Model 1 incorporated seven predictors, including gender, age, smoking, self-report health condition, BMI, depressive symptoms, and cognitive function. Besides, five physical performance-based models were established based on Model 1, adding handgrip strength (Model 2), SPPB (Model 3), gait speed (Model 4), handgrip strength plus SPPB (Model 5), and handgrip strength plus gait speed (Model 6), respectively. Model 1 to Model 6 are presented in purple, dark blue, red, yellow, orange, bright blue lines, respectively

**Fig. 2:**
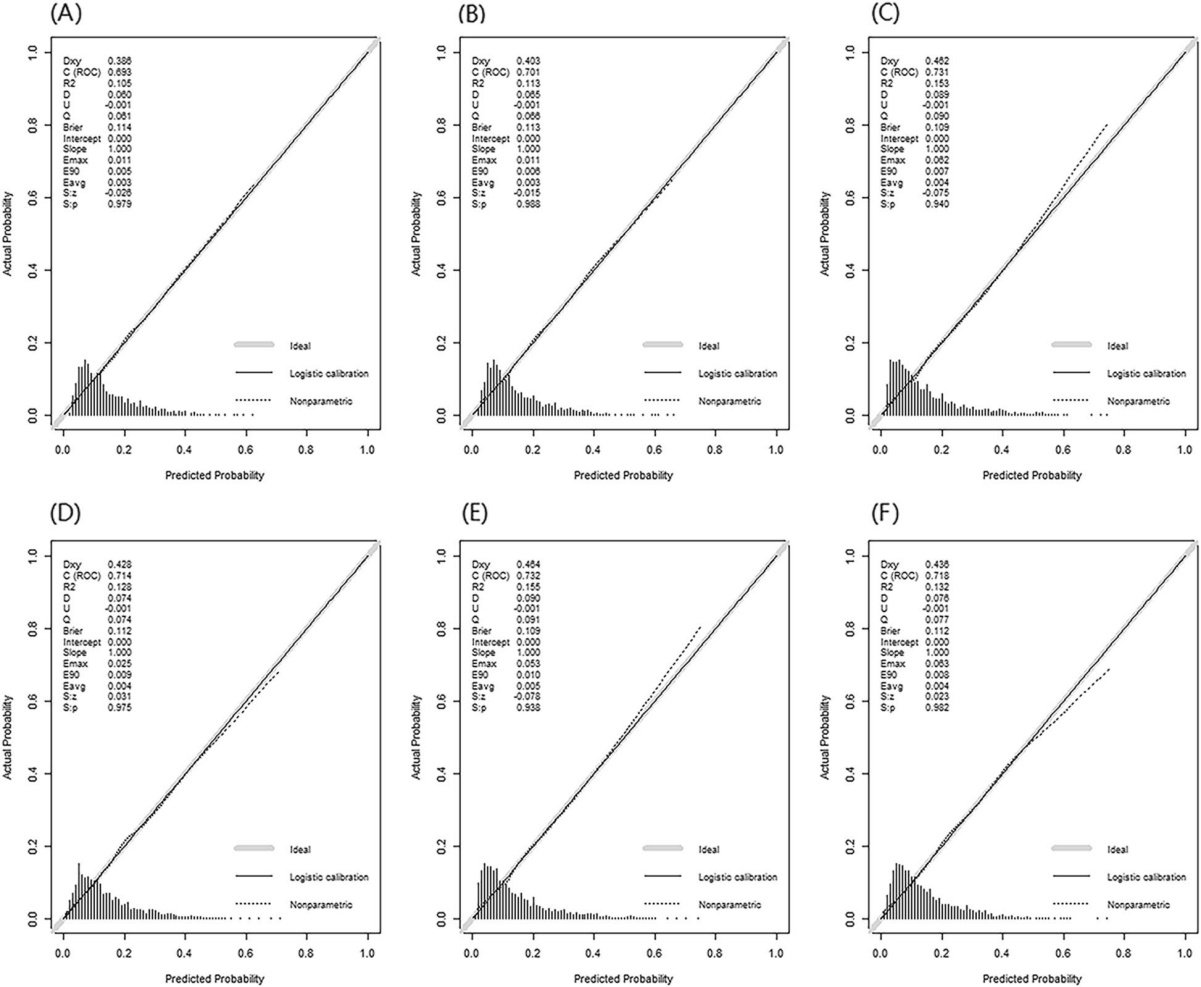
Calibration curves for different models. **A** Model 1, (**B**) Model 2, (**C**) Model 3, (**D**) Model 4, (**E**) Model 5, and (**F**) Model 6. Note. Model 1 incorporated seven predictors, including gender, age, smoking, self-report health condition, BMI, depressive symptoms, and cognitive function. Besides, five physical performance-based models were established based on Model 1, adding handgrip strength (Model 2), SPPB (Model 3), gait speed (Model 4), handgrip strength plus SPPB (Model 5), and handgrip strength plus gait speed (Model 6), respectively. Model-predicted probability and actual probability for ADL disability among older adults were plotted in the x- and y-axis, respectively. The diagonal gray line represents an ideal plot for the calibration plot. The solid black line represents the performance of the prediction model, of which a closer match to the diagonal gray line indicates a better calibration

**Fig. 3:**
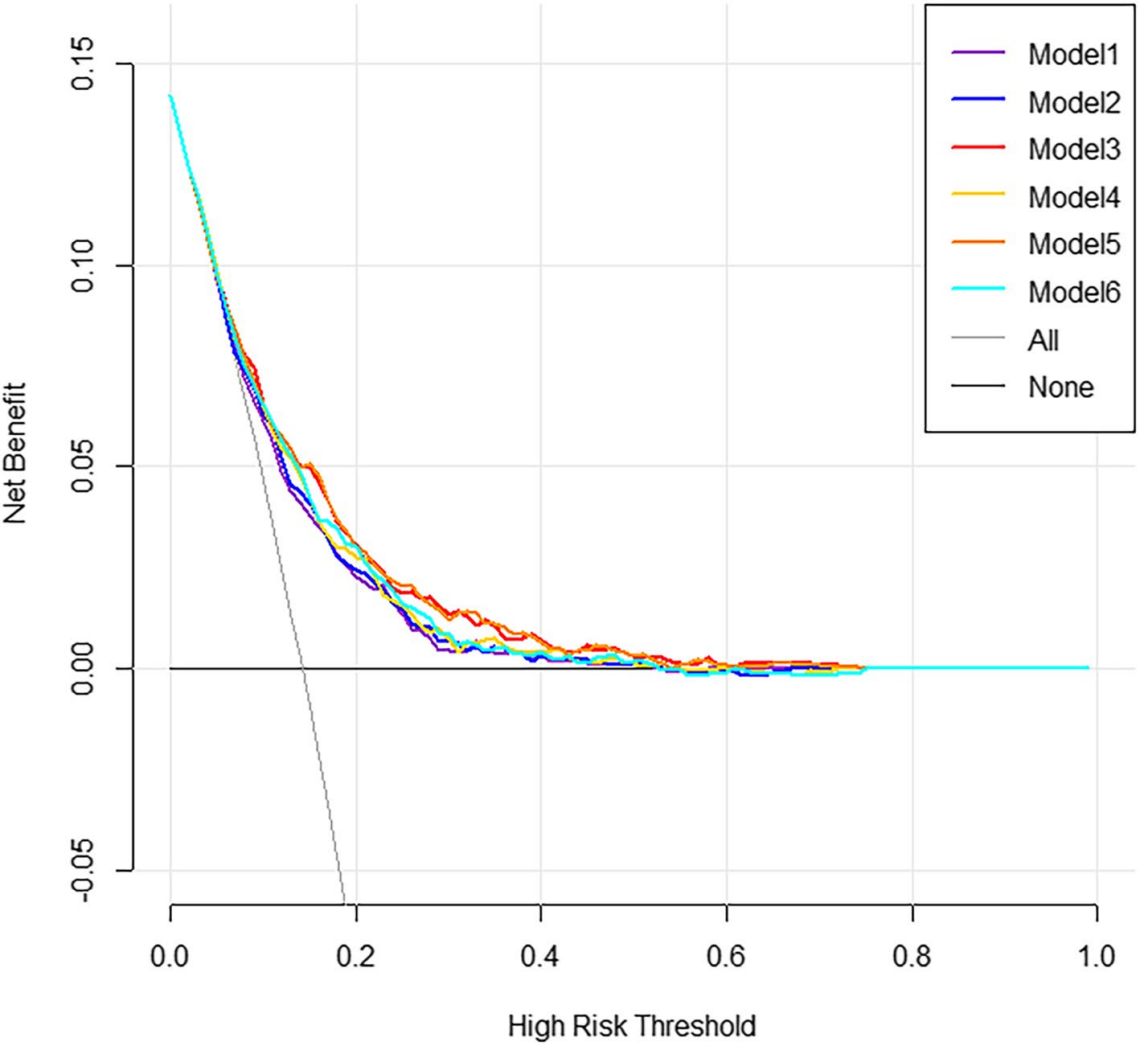
The Decision curves analysis for different prediction models. Note. Model 1 incorporated seven predictors, including gender, age, smoking, self-report health condition, BMI, depressive symptoms, and cognitive function. Besides, five physical performance-based models were established based on Model 1, adding handgrip strength (Model 2), SPPB (Model 3), gait speed (Model 4), handgrip strength plus SPPB (Model 5), and handgrip strength plus gait speed (Model 6), respectively. Model 1 to Model 6 are presented in purple, dark blue, red, yellow, orange, bright blue lines, respectively. Y-axis indicates net benefit, calculated by summing the benefits (true positives) and subtracting the harms (false positives). The straight line represents the assumption that all participants will develop ADL disability, and the horizontal line represents the assumption that no participants will develop ADL disability

The results of six multivariate logistic regression models were shown in Table S3. Nomograms were built to visualize the models and for convenient use (Fig. S2, S3, S4, S5, S6, Fig. S7). Compared to Model 1 (AUC = 0.693, C-index = 0.693, 95%CI = 0.661–0.725), all five physical performance-based models had better discrimination with greater C-index (Fig. 1**,** Table 2). Among these five models, the comprehensive model (Model 5 handgrip strength + SPPB model: AUC = 0.732, C-index = 0.732, 95%CI = 0.702–0.763) had the best discrimination, very close to that of the lower limb model (Model 3 SPPB model: AUC = 0.731, C-index = 0.731, 95%CI = 0.701–0.762), and significantly better than upper limb model (Model 2 handgrip strength model: AUC = 0.701, C-index = 0.701, 95%CI = 0.669–0.733). Replacing SPPB with gait speed, the discriminations of the models attenuated slightly (Model 4 gait speed model: AUC = 0.714, C-index = 0.714, 95%CI = 0.684–0.745; Model 6 handgrip strength + gait speed model: AUC = 0.718, C-index = 0.718, 95%CI = 0.687–0.749), but were still better than upper limb model (Model 2 handgrip strength model). Calibration plots of all six models demonstrated that the points were close to the 45-degree line, indicating good calibration (Fig. 2). Furthermore, a DCA analysis was used to compare the clinical utility as seen in Fig. 3, the comprehensive models (Model 5: handgrip strength + SPPB model) and lower limb model (Model 3: SPPB model) have similar net benefits and are significantly better than upper limb model (Model 2: handgrip strength model). Correspondingly, when replacing SPPB with gait speed, the clinical utility of the lower limb models (Model 4: gait speed model) and the comprehensive models (Model 6: handgrip strength + gait speed model) attenuated slightly, but were still better than upper limb model (Model 2: handgrip strength model).

**Table 2:** Comparison of C-index between different models

	C-index (95%CI)	*P* value
Model 1	0.693 (0.661–0.725)	<0.001^*^
Model 2	0.701 (0.669–0.733)	<0.001^*^
Model 3	0.731 (0.701–0.762)	<0.001^*^
Model 4	0.714 (0.684–0.745)	<0.001^*^
Model 5	0.732 (0.702–0.763)	<0.001^*^
Model 6	0.718 (0.687–0.749)	<0.001^*^

*Note*. *C-index* concordance index

Model 1 incorporated seven predictors, including gender, age, smoking, self-report health condition, BMI, depressive symptoms, and cognitive function. Besides, five physical performance- based models were established based on Model 1, adding handgrip strength (Model 2), SPPB (Model 3), gait speed (Model 4), handgrip strength plus SPPB (Model 5), and handgrip strength plus gait speed (Model 6), respectively

*p < 0.05

The predictive performance of each model was internally validated by conducting 1000 bootstrap resamples. The bootstrap-corrected C-index of Model 1 to Model 6 were 0.674, 0.682, 0.716, 0.697, 0.716, and 0.700, respectively (data not shown). The bootstrap-corrected calibration plots (Fig. S8) showed that the comprehensive models (Model 5:handgrip strength + SPPB model) and lower limb model (Model 3: SPPB model) had similar calibration and were significantly better than upper limb model (Model 2: handgrip strength model). Replacing SPPB with gait speed, the calibration of the lower limb model (Model 4: gait speed model) had no obvious change.

### Incremental benefit analysis

NRI and IDI were used to estimate the added value of the extended model (Table 3). Not surprisingly, all five physical performance-based models had significant improvement in discrimination and reclassification compared with Model 1 (all NRI and IDI > 0 and *P* < 0.05). Additionally, when adding lower limb indexes (regardless of gait speed or SPPB) to the upper limb model (Model 2: handgrip strength model), significantly incremental benefits of subsequent extended models (Model 5 handgrip strength + SPPB model and Model 6 handgrip strength + gait speed model) were observed (Model 2 vs Model 5: NRI = 0.358, 95%CI = 0.239–0.476; IDI = 0.031, 95%CI = 0.021–0.040; Model 2 vs Model 6: NRI = 0.346, 95%CI = 0.229–0.463; IDI = 0.012, 95%CI = 0.007–0.017). In contrast, when upper limb indexes were added to the lower limb model (Model 3 SPPB model and Model 4 gait speed model), no obvious improvement was identified in the extended models (Model 5 handgrip strength + SPPB model and Model 6 handgrip strength + gait speed model) with non-statistically significant effect (Model 3 vs Model 5: NRI = 0.058, 95%CI = 0.062–0.178; IDI = 0.001, 95%CI = 0.010–0.023; Model 4 vs Model 6: NRI = 0.071, 95%CI = 0.049–0.191; IDI = 0.003, 95%CI = 0.000–0.006). The above results suggested the lower limb models may play a critical role in the prediction of ADL disability, whereas the upper limb model may not.

**Table 3:** NRI and IDI between different models

	NRI (95%CI)	*P* value	IDI (95%CI)	*P* value
Model 1 vs Model 2	0.146 (0.026–0.265)	0.017^*^	0.005 (0.001–0.009)	0.011^*^
Model 1 vs Model 3	0.406 (0.288–0.524)	< 0.001^*^	0.035 (0.024–0.045)	< 0.001^*^
Model 1 vs Model 4	0.367 (0.251–0.484)	< 0.001^*^	0.014 (0.008–0.019)	< 0.001^*^
Model 1 vs Model 5	0.396 (0.278–0.514)	< 0.001^*^	0.036 (0.025–0.046)	< 0.001^*^
Model 1 vs Model 6	0.341 (0.224–0.459)	< 0.001^*^	0.017 (0.010–0.023)	< 0.001^*^
Model 2 vs Model 5	0.358 (0.239–0.476)	< 0.001^*^	0.031 (0.021–0.040)	< 0.001^*^
Model 2 vs Model 6	0.346 (0.229–0.463)	< 0.001^*^	0.012 (0.007–0.017)	< 0.001^*^
Model 3 vs Model 5	0.058 (0.062–0.178)	0.341	0.001 (0.010–0.023)	0.276
Model 4 vs Model 6	0.071 (0.049–0.191)	0.247	0.003 (0.000–0.006)	0.049

*Note*. *IDI* Integrated Discrimination Improvement, *NRI* Net Reclassification Improvement; Model 1 incorporated seven predictors, including gender, age, smoking, self-report health condition, BMI, depressive symptoms, and cognitive function. Besides, five physical performance-based models were established based on Model 1, adding handgrip strength (Model 2), SPPB (Model 3), gait speed (Model 4), handgrip strength plus SPPB (Model 5), and handgrip strength plus gait speed (Model 6), respectively

*p < 0.05

## Discussion

In this study, we developed five extended prediction models by adding different physical performances—handgrip strength, SPPB, gait speed, handgrip strength plus SPPB, and handgrip strength plus gait speed, and internally validated the models by C-index, calibration plots, DCA analysis, NRI and IDI. We found that all five novel models, as compared to the fundamental model, demonstrated better predictive performance in accuracy, discrimination, and clinical utility, manifested as improved NRI and IDI. In addition, we observed the prediction of ADL disability was largely contributed by the lower limb model, where the upper limb model contributed little**.** Furthermore, considering clinical applications, we also explored the possibility of simplifying the lower limb model by replacing SPPB with gait speed and found that the predictive value of the models attenuated slightly, but still better than the upper limb model.

Previous studies have reported the predictive value of physical performances for ADL disability, indicating handgrip strength, SPPB score, and gait speed were reliable prediction tools [9–12, 42]. However, few studies have considered including the physical performances into the prediction models. Cristina Minneci et al. [12] developed four prediction models incorporating physical performances to predict ADL disability, yet without model validation. Jack M. Guralnik [10] and Wen-Ni Wennie Huang [9] used different methods to construct physical performance-based models for the prediction of ADL disability. However, both studies had the limitation that the models were validated only by discrimination, ignoring calibration and clinical utility. Recently, Nini H. Jonkman [11] developed a model based on handgrip strength, gait speed, repeated chair stands test, and other traditional risk factors to predict ADL disability using four European cohort studies, and they applied internal-external cross-validation to assess the model by discrimination and calibration. The performance of the model determines the clinical application, so it is necessary to evaluate and validate the model comprehensively and extensively. Our study extended the prediction model by adding physical performances and validated the model by C-index, calibration plots, DCA analysis, NRI and IDI. After a comprehensive assessment, our updated models had better overall performance for predicting ADL disability.

It is noteworthy that, in our study, we observed that the lower limb models may play a critical role in the prediction of ADL disability, whereas the upper limb model may not. A recent study comprised 1591 adults aged ≥65 years from the Sasaguri Genkimon Study (SGS), also observed better discrimination of gait speed models (C index = 0.778, 95%CI = 0.759–0.803) than handgrip strength models (C index = 0.775, 95%CI = 0.756–0.800) on the prediction of functional disability risk among older adults [19]. Another study indicated that the gait speed test could better discriminate ADL disability compared with hand-grip strength both in male (handgrip strength: AUC = 0.67, 95%CI = 0.63–0.72; gait speed: AUC = 0.70, 95%CI = 0.66–0.74) and female (handgrip strength: AUC = 0.64, 95%CI = 0.59–0.68; gait speed: AUC = 0.68, 95%CI = 0.64–0.72) as well [32]. It was therefore, not surprised to identify that lower limb models had comparable predictive value to the comprehensive models combing upper and lower limb. It seems reasonable and sufficient to use only the lower limb model to predict ADL disability among Chinese older population in clinical practice. To further simplify the lower limb model, we replaced SPPB with gait speed and found nuanced difference. This has practical implications concerning the feasibility. For older adults with poor physical tolerance or in situations with time constraints, we assumed that the model including gait speed, a more practical and simpler tool, would be handier than the SPPB model. Moreover, the gait speed model could also be an effective tool as the first step in screening a large number of older adults to identify and recruit into clinical trials participants with a specific level of functioning [10].

Apart from physical performances, our updated model yielded the following independent risk factors: gender, age, smoking, self-report health condition, BMI, depressive symptoms, and cognitive function. Older age, smoking, overweight or underweight, poor self-reported health conditions, or poor mental health are commonly associated with ADL disability [30, 31]. Most modifiable risk factors may reverse the decline of ADL disability and easy-to-measure variables that discriminate well in predicting functional decline in community-dwelling older adults. Clinicians can utilize this set of variables to screen individuals on their risk of functional decline in the future. Therefore, prevention and intervention strategies should focus on guiding older adults to develop a healthy lifestyle and improving their physical and mental fitness, especially in the older male population.

Early prediction and prevention of disability should be a priority for healthy aging. Available evidence suggests that one-size-fits-all preventive interventions for ADL disability are unsuccessful because of the heterogeneity of older adults [43]. For example, two older adults with the same risk probability of ADL disability may suffer from various risk factors, so their care needs and intervention strategies should also be tailored depending on their specific situation. The nomogram provides individualized identification for older adults at risk. Health care workers could make targeted interventions according to the scores of different items on nomogram for each subject, improving the efficiency of interventions [28, 44]. In addition, we would like to emphasize that all predictors we include could be measured in the real-world clinical setting. Particularly, we chose physical performances into the prediction tool, providing more objective and steady information than self-report measures. At the same time, many technology-based devices, such as force platforms, wearable devices, and accelerometer sensors, have been designed to collect the information of physical performances. Nomograms could be applied to information and communication technology (ICT) to closely monitor the health status of older adults. For example, wearable devices such as smart bracelets could be used to collect information about their physical performances. With the information collected, physicians can use the nomogram to calculate the probabilities for ADL disability. This strategy, combining the nomogram with the use of ICT, provides new ideas and methods for the efficient intervention of older adults. The community could carry out physical performance screening for older adults using physical performance-based models, especially lower limb models, to predict the probability of ADL disability among elders. It would be helpful to avoid or delay the occurrence of ADL disability, and improve the quality of life of older adults.

However, the nomogram should be interpreted with caution with reference to statistical results such as the 95%CI of ORs and *P* values. For example, although the lowest risk for ADL disability seems to be among heavy smokers who smoke more than 20/day – even lower than for those who have never smoked in our nomograms, this result is non-statistically significant in our models according to the 95%CI of ORs and *P* values (*P* values > 0.05). There might be two possible interpretations. First, we assumed that the participants who smoked more than 20 cigarettes/day at baseline may be healthier than those who smoked less than 20/day or non-smokers. In other words, if an individual’s health were hampered, he/she may have a lower likelihood of heavy smoking. Also, Margare R. Becklake et al. proposed the concept of “healthy smoker” [45]. They found that smokers have healthier lung function that is relatively resistant to the effects of smoking. Second, smoking is a modifiable factor. During the 4-year follow-up period, participants who smoked more than 20 cigarettes/day at baseline were likely to have been forced to quit or reduce smoking in 2015 due to other medical conditions like respiratory diseases or others. And ADL disability is also sometimes a reversible event. Therefore, future repeated exposure measures are warranted.

Our study has several limitations. First, we didn’t directly compare our model against the prediction models of previous studies. Such comparison was not easy to conduct because not all the variables required by these models are collected in CHARLS. However, we included as many traditional risk factors related to ADL disability in previous studies as possible and conducted a fundamental model based only on these variables to evaluate the added value of our updated model. Second, there is large heterogeneity in the baseline characteristics of the excluded and included group, which may bias our results; Last but not least, although internal validation adequately assesses the performance of the model, external validation in another cohort waits for further efforts. It is still unclear whether the model developed in this study can be used by all community older adults. Therefore, further prospective multicentered validation studies are warranted to support our study.

In conclusion, we have developed novel physical performance-based models with improved predictive values to assess the risk of ADL disability among Chinese older adults. These novel models, lower limb models in particular, achieved satisfactory performance in internal validation. Nonetheless, further multicentered external validation studies are necessary. Overall, the application of the updated models will better inform physicians of the risk of ADL disability and guide the targeted interventions.

## Supplementary Information

**Additional file 1: Table S1**. The baseline characteristics of participants with participants excluded vs. included at baseline.

**Additional file 2: Table S2**. The baseline characteristics of participants with complete outcome vs. missing outcome at follow-up.

**Additional file 3: Table S3.** Prediction models of ADL established by logistic regression analysis.

**Additional file 4: Figure S1**.Study flow.

**Additional file 5: Figure S2.** Nomogram for Model 1(fundamental model).

**Additional file 6: Figure S3.** Nomogram for Model 2 (handgrip strength model).

**Additional file 7: Figure S4.** Nomogram for Model 3(SPPB model).

**Additional file 8: Figure S5.** Nomogram for Model 4(gait speed model).

**Additional file 9: Figure S6.** Nomogram for Model 5 (handgrip strength + SPPB model).

**Additional file 10: Figure S7.** Nomogram for Model 6(handgrip strength + gait speed model).

**Additional file 11: Figure S8.** Bootstrap corrected calibration curves.

## Data Availability

The datasets generated and analyzed during the current study are available in the CHARLS website, available in http://charls.pku.edu.cn/en.

## Abbreviations

ADL: Activities of daily living
SPPB: Short Physical Performance Battery
CHARLS: China Health and Retirement Longitudinal Study
BMI: Body mass index
CES-D-10: Center for Epidemiologic Studies Depression Scale-10 items
AIC: Akaike information criterion
C index: Concordance index
IDI: Integrated Discrimination Improvement
NRI: Net Reclassification Improvement
OR: Odds ratio
95%CI: 95% confidential interval
AUC: Area under the receiver-operating characteristic curve
DCA: Clinical decision curve analysis
ICT: Information and communication technology

## References

[1] Abdulraheem IS, Oladipo AR, Amodu MO (2011). Prevalence and correlates of physical disability and functional limitation among elderly rural population in Nigeria. J Aging Res.

[2] Millan-Calenti JC, Tubio J, Pita-Fernandez S, Gonzalez-Abraldes I, Lorenzo T, Fernandez-Arruty T (2010). Prevalence of functional disability in activities of daily living (ADL), instrumental activities of daily living (IADL) and associated factors, as predictors of morbidity and mortality. Arch Gerontol Geriatr.

[3] Pereira C, Bravo J, Raimundo A, Tomas-Carus P, Mendes F, Baptista F (2020). Risk for physical dependence in community-dwelling older adults: the role of fear of falling, falls and fall-related injuries. Int J Older People Nursing.

[4] McGrath R, Vincent BM, Hackney KJ, Al Snih S, Graham J, Thomas L (2020). Weakness and cognitive impairment are independently and jointly associated with functional decline in aging Americans. Aging Clin Exp Res.

[5] Liu H, Jiao J, Zhu C, Zhu M, Wen X, Jin J (2020). Potential associated factors of functional disability in Chinese older inpatients: a multicenter cross-sectional study. BMC Geriatr.

[6] Vermeulen J, Neyens JC, van Rossum E, Spreeuwenberg MD, de Witte LP (2011). Predicting ADL disability in community-dwelling elderly people using physical frailty indicators: a systematic review. BMC Geriatr.

[7] Raina P, Gilsing A, Mayhew AJ, Sohel N, van den Heuvel E, Griffith LE (2020). Individual and population level impact of chronic conditions on functional disability in older adults. PLoS One.

[8] Shi Z, Lin J, Xiao J, Fang Y (2021). Sex differences in the association between latent class of lifestyle and disability among older adults in China. BMC Geriatr.

[9] Wennie Huang WN, Perera S, VanSwearingen J, Studenski S (2010). Performance measures predict onset of activity of daily living difficulty in community-dwelling older adults. J Am Geriatr Soc.

[10] Guralnik JM, Ferrucci L, Pieper CF, Leveille SG, Markides KS, Ostir GV (2000). Lower extremity function and subsequent disability: consistency across studies, predictive models, and value of gait speed alone compared with the short physical performance battery. J Gerontol A Biol Sci Med Sci.

[11] Jonkman NH, Colpo M, Klenk J, Todd C, Hoekstra T, Del Panta V (2019). Development of a clinical prediction model for the onset of functional decline in people aged 65-75 years: pooled analysis of four European cohort studies. BMC Geriatr.

[12] Minneci C, Mello AM, Mossello E, Baldasseroni S, Macchi L, Cipolletti S (2015). Comparative study of four physical performance measures as predictors of death, incident disability, and falls in unselected older persons: the insufficienza Cardiaca negli Anziani Residenti a Dicomano study. J Am Geriatr Soc.

[13] Elboim-Gabyzon M, Danial-Saad A. Correlation between the ability to manipulate a touchscreen device and hand strength and manual dexterity among community-living older individuals. Int J Environ Res Public Health. 2021;18(17). 10.3390/ijerph18179408. 10.3390/ijerph18179408 PMC8431526 34501994

[14] Ostir GV, Markides KS, Black SA, Goodwin JS (1998). Lower body functioning as a predictor of subsequent disability among older Mexican Americans. J Gerontol A Biol Sci Med Sci.

[15] Fortes SQ, Aliberti MJR, Apolinario D, Melo-Fortes JA, Sitta MC, Jacob W (2020). Role of gait speed, strength, and balance in predicting adverse outcomes of acutely ill older outpatients. J Nutr Health Aging.

[16] Giampaoli S, Ferrucci L, Cecchi F, Lo Noce C, Poce A, Dima F (1999). Hand-grip strength predicts incident disability in non-disabled older men. Age Ageing.

[17] Shinkai S, Watanabe S, Kumagai S, Fujiwara Y, Amano H, Yoshida H (2000). Walking speed as a good predictor for the onset of functional dependence in a Japanese rural community population. Age Ageing.

[18] Guralnik JM, Simonsick EM, Ferrucci L, Glynn RJ, Berkman LF, Blazer DG (1994). A short physical performance battery assessing lower extremity function: association with self-reported disability and prediction of mortality and nursing home admission. J Gerontol.

[19] Chen T, Honda T, Chen S, Kishimoto H, Kumagai S, Narazaki K (2021). Potential utility of physical function measures to improve the risk prediction of functional disability in community-dwelling older Japanese adults: a prospective study. BMC Geriatr.

[20] den Ouden ME, Schuurmans MJ, Mueller-Schotte S, van der Schouw YT (2013). Identification of high-risk individuals for the development of disability in activities of daily living. A ten-year follow-up study. Exp Gerontol.

[21] Zhao YH, Hu YS, Smith JP, Strauss J, Yang GH (2014). Cohort profile: the China health and retirement longitudinal study (CHARLS). Int J Epidemiol.

[22] Katz S, Ford AB, Moskowitz RW, Jackson BA, Jaffe MW (1963). Studies of illness in the aged. The index of Adl: a standardized measure of biological and psychosocial function. JAMA.

[23] Zhong Y, Wang J, Nicholas S (2017). Gender, childhood and adult socioeconomic inequalities in functional disability among Chinese older adults. Int J Equity Health.

[24] Roberts HC, Denison HJ, Martin HJ, Patel HP, Syddall H, Cooper C (2011). A review of the measurement of grip strength in clinical and epidemiological studies: towards a standardised approach. Age Ageing.

[25] Zuo M, Gan C, Liu T, Tang J, Dai J, Hu X (2019). Physical predictors of cognitive function in individuals with hypertension: evidence from the CHARLS Basline survey. West J Nurs Res.

[26] Zhong BX, Zhong HL, Zhou GQ, Xu WQ, Lu Y, Zhao Q (2021). Physical performance and risk of hip fracture in community-dwelling elderly people in China: a 4-year longitudinal cohort study. Maturitas.

[27] Wu X, Li X, Xu M, Zhang Z, He L, Li Y (2021). Sarcopenia prevalence and associated factors among older Chinese population: findings from the China health and retirement longitudinal study. PLoS One.

[28] Zhang L, Cui HJ, Chen QZ, Li Y, Yang CX, Yang YF (2021). A web-based dynamic Nomogram for predicting instrumental activities of daily living disability in older adults: a nationally representative survey in China. BMC Geriatr.

[29] Arnau A, Espaulella J, Serrarols M, Canudas J, Formiga F, Ferrer M (2016). Risk factors for functional decline in a population aged 75 years and older without total dependence: a one-year follow-up. Arch Gerontol Geriatr.

[30] Chen S, Qin J, Li Y, Wei Y, Long B, Cai J, et al. Disability and its influencing factors among the elderly in a county, Guangxi Province, China. Int J Environ Res Public Health. 2018;15(9). 10.3390/ijerph15091967. 10.3390/ijerph15091967 PMC6163965 30205622

[31] Li ZH, Chen Q, Byers Kraus V, Shen D, Zhang XR, Zhang PD (2020). Leisure activities and disability in activities of daily living among the oldest-old Chinese population: evidence from the Chinese longitudinal healthy longevity study. Aging (Albany N Y).

[32] Zhang L, Guo L, Wu H, Gong X, Lv J, Yang Y (2019). Role of physical performance measures for identifying functional disability among Chinese older adults: data from the China health and retirement longitudinal study. PLoS One.

[33] Fu LY, Wang XX, Wu X, Li B, Huang LL, Li BB (2018). Association between obesity and sickness in the past two weeks among middle-aged and elderly women: a cross-sectional study in southern China. PLoS One.

[34] Wang J, Zhu WH, Li YF, Zhu WW (2020). Interaction between worsening cognitive function and deteriorating functional status on depressive symptoms among Chinese community-dwelling elders. Geriatr Gerontol Int.

[35] Ye X, Zhu D, Chen S, He P (2020). The association of hearing impairment and its severity with physical and mental health among Chinese middle-aged and older adults. Health Qual Life Outcomes.

[36] Akobeng AK (2007). Understanding diagnostic tests 3: receiver operating characteristic curves. Acta Paediatr.

[37] Lee MC, Hsu CC, Tsai YF, Chen CY, Lin CC, Wang CY (2018). Criterion-referenced values of grip strength and usual gait speed using instrumental activities of daily living disability as the criterion. J Geriatr Phys Ther.

[38] Zhang JX, Song W, Chen ZH, Wei JH, Liao YJ, Lei J (2013). Prognostic and predictive value of a microRNA signature in stage II colon cancer: a microRNA expression analysis. Lancet Oncol.

[39] Vickers AJ, Cronin AM, Elkin EB, Gonen M. Extensions to decision curve analysis, a novel method for evaluating diagnostic tests, prediction models and molecular markers. BMC Med Inform Decis Mak. 2008;8. 10.1186/1472-6947-8-53. 10.1186/1472-6947-8-53 PMC2611975 19036144

[40] Nead KT, Zhou MJ, Caceres RD, Sharp SJ, Wehner MR, Olin JW (2013). Usefulness of the addition of beta-2-microglobulin, cystatin C and C-reactive protein to an established risk factors model to improve mortality risk prediction in patients undergoing coronary angiography. Am J Cardiol.

[41] Lewandowska M, Wieckowska B, Sajdak S, Lubinski J. Pre-pregnancy obesity vs. other risk factors in probability models of preeclampsia and gestational hypertension. Nutrients. 2020;12(9). 10.3390/nu12092681. 10.3390/nu12092681 PMC7551880 32887442

[42] Wang DXM, Yao J, Zirek Y, Reijnierse EM, Maier AB (2020). Muscle mass, strength, and physical performance predicting activities of daily living: a meta-analysis. J Cachexia Sarcopenia Muscle.

[43] Bleijenberg N, Zuithoff NPA, Smith AK, de Wit NJ, Schuurmans MJ (2017). Disability in the individual ADL, IADL, and mobility among older adults: a prospective cohort study. J Nutr Health Aging.

[44] Park SY (2018). Nomogram: an analogue tool to deliver digital knowledge. J Thorac Cardiovasc Surg.

[45] Becklake MR, Lalloo U (1990). The 'healthy smoker': a phenomenon of health selection?. Respiration.

