# Supplementary material for: Novel physical performance-based models for activities of daily living disability prediction among Chinese older community population: a nationally representative survey in China
Source: BMC Geriatr. 2022 Mar 31;22:267. doi: 10.1186/s12877-022-02905-y (PMC8974010; doi:10.1186/s12877-022-02905-y)
Supplement: Supplementary file 1 — Additional file 1: Table S1. The baseline characteristics of participants with participants excluded vs. included at baseline. [file 12877_2022_2905_MOESM1_ESM.doc]

**Table A.1** The baseline characteristics of participants with participants excluded vs. included at baseline

| Variables | **Baseline Data** | | |
| --- | --- | --- | --- |
|  | Full data | Missing data | *P* value |
| Overall | ***N=4303*** | ***N=2840*** |  |
| Gender: |  |  | **<0.001*** |
| Male | 1954 (45.4%) | 1665 (58.6%) |  |
| Female | 2349 (54.6%) | 1175 (41.4%) |  |
| Age: |  |  | **<0.001*** |
| 60-64 | 1549 (36.0%) | 1223 (43.1%) |  |
| 65-69 | 1066 (24.8%) | 670 (23.6%) |  |
| 70-74 | 824 (19.1%) | 444 (15.6%) |  |
| >=75 | 864 (20.1%) | 503 (17.7%) |  |
| Hukou: |  |  | **<0.001*** |
| Agricultural | 3468 (80.6%) | 1925 (68.0%) |  |
| Non-Agricultural | 835 (19.4%) | 906 (32.0%) |  |
| Education: |  |  | **<0.001*** |
| Illiterate | 1725 (40.1%) | 841 (29.7%) |  |
| Primary school | 1948 (45.3%) | 1212 (42.9%) |  |
| Middle school | 427 (9.92%) | 453 (16.0%) |  |
| High school | 142 (3.30%) | 226 (7.99%) |  |
| College and above | 61 (1.42%) | 95 (3.36%) |  |
| Marital status: |  |  | **<0.001*** |
| Married/Cohabitated | 2285 (76.5%) | 2312 (81.6%) |  |
| widowed | 644 (21.6%) | 447 (15.8%) |  |
| Other | 58 (1.94%) | 76 (2.68%) |  |
| Social activity: |  |  | **0.001*** |
| Never | 2314 (53.8%) | 1182 (50.6%) |  |
| Not regularly | 512 (11.9%) | 238 (10.2%) |  |
| Almost Weekly | 418 (9.71%) | 252 (10.8%) |  |
| Almost daily | 1059 (24.6%) | 666 (28.5%) |  |
| Self-report health: |  |  | **<0.001*** |
| Good | 389 (9.04%) | 547 (19.6%) |  |
| Fair | 1217 (28.3%) | 939 (33.7%) |  |
| Poor | 1734 (40.3%) | 908 (32.6%) |  |
| Very poor | 963 (22.4%) | 394 (14.1%) |  |
| Smoking: |  |  | **0.003*** |
| Never | 2796 (65.0%) | 1518 (63.6%) |  |
| Quit | 579 (13.5%) | 273 (11.4%) |  |
| Less than 20 /day | 445 (10.3%) | 266 (11.2%) |  |
| More than 20 /day | 483 (11.2%) | 328 (13.8%) |  |
| Drinking: |  |  | **<0.001*** |
| Never | 2636 (61.3%) | 1504 (54.2%) |  |
| Quit | 492 (11.4%) | 289 (10.4%) |  |
| Less than once/month | 263 (6.11%) | 221 (7.96%) |  |
| More than once/month | 912 (21.2%) | 763 (27.5%) |  |
| Night sleep: |  |  | **<0.001*** |
| 6-9 | 2356 (54.8%) | 1470 (62.4%) |  |
| <6 | 1596 (37.1%) | 654 (27.8%) |  |
| >=9 | 351 (8.16%) | 231 (9.81%) |  |
| Comorbidity: |  |  | **<0.001*** |
| 0 | 910 (21.1%) | 976 (35.0%) |  |
| 1 | 1245 (28.9%) | 837 (30.0%) |  |
| >=2 | 2148 (49.9%) | 975 (35.0%) |  |
| Gait speed | 0.59 [0.45;0.74] | 0.68 [0.55;0.84] | **<0.001*** |
| BMI: |  |  | **<0.001*** |
| Normal | 2300 (53.5%) | 812 (60.1%) |  |
| Underweight | 494 (11.5%) | 99 (7.33%) |  |
| Overweight | 1101 (25.6%) | 353 (26.1%) |  |
| obese | 408 (9.48%) | 87 (6.44%) |  |
| Depressive symptoms: |  |  | **<0.001*** |
| Normal | 2365 (55.0%) | 1480 (63.9%) |  |
| depression | 1938 (45.0%) | 836 (36.1%) |  |
| Cognitive function | 9.00 [5.00;12.5] | 11.0 [7.38;14.0] | **<0.001*** |
| SPPB | 8.00 [6.00;10.0] | 9.00 [7.00;10.0] | **<0.001*** |

*Note. ADLs = activities of daily living, BMI= body mass index, SPPB= Short Physical Performance Battery;*

********p < 0.05*

**Table A.2** The baseline characteristics of participants with complete outcome vs. missing outcome at follow-up

| Variables | 4-year follow-up | | |
| --- | --- | --- | --- |
|  | Missing data | Full data | *P* value |
| Overall | *N=2111* | *N=2192* |  |
| Gender: |  |  | **<0.001*** |
| Male | 1062 (50.3%) | 892 (40.7%) |  |
| Female | 1049 (49.7%) | 1300 (59.3%) |  |
| Age: |  |  | **0.002*** |
| 60-64 | 740 (35.1%) | 809 (36.9%) |  |
| 65-69 | 497 (23.5%) | 569 (26.0%) |  |
| 70-74 | 401 (19.0%) | 423 (19.3%) |  |
| >=75 | 473 (22.4%) | 391 (17.8%) |  |
| Hukou: |  |  | **0.007*** |
| Agricultural | 1666 (78.9%) | 1802 (82.2%) |  |
| Non-Agricultural | 445 (21.1%) | 390 (17.8%) |  |
| Education: |  |  | 0.151 |
| Illiterate | 814 (38.6%) | 911 (41.6%) |  |
| Primary school | 975 (46.2%) | 973 (44.4%) |  |
| Middle school | 213 (10.1%) | 214 (9.76%) |  |
| High school | 72 (3.41%) | 70 (3.19%) |  |
| College and above | 37 (1.75%) | 24 (1.09%) |  |
| Marital status: |  |  | 0.830 |
| Married/Cohabitated | 605 (76.1%) | 1680 (76.6%) |  |
| widowed | 176 (22.1%) | 468 (21.4%) |  |
| Other | 14 (1.76%) | 44 (2.01%) |  |
| Social activity: |  |  | 0.689 |
| Never | 1151 (54.5%) | 1163 (53.1%) |  |
| Not regularly | 251 (11.9%) | 261 (11.9%) |  |
| Almost Weekly | 206 (9.76%) | 212 (9.67%) |  |
| Almost daily | 503 (23.8%) | 556 (25.4%) |  |
| Self-report health: |  |  | 0.925 |
| Good | 189 (8.95%) | 200 (9.12%) |  |
| Fair | 601 (28.5%) | 616 (28.1%) |  |
| Poor | 857 (40.6%) | 877 (40.0%) |  |
| Very poor | 464 (22.0%) | 499 (22.8%) |  |
| Smoking: |  |  | **0.003*** |
| Never | 1314 (62.2%) | 1482 (67.6%) |  |
| Quit | 311 (14.7%) | 268 (12.2%) |  |
| Less than 20 /day | 233 (11.0%) | 212 (9.67%) |  |
| More than 20 /day | 253 (12.0%) | 230 (10.5%) |  |
| Drinking: |  |  | **<0.001*** |
| Never | 1214 (57.5%) | 1422 (64.9%) |  |
| Quit | 255 (12.1%) | 237 (10.8%) |  |
| Less than once/month | 147 (6.96%) | 116 (5.29%) |  |
| More than once/month | 495 (23.4%) | 417 (19.0%) |  |
| Night sleep: |  |  | 0.447 |
| 6-9 | 1140 (54.0%) | 1216 (55.5%) |  |
| <6 | 789 (37.4%) | 807 (36.8%) |  |
| >=9 | 182 (8.62%) | 169 (7.71%) |  |
| Comorbidity: |  |  | 0.852 |
| 0 | 439 (20.8%) | 471 (21.5%) |  |
| 1 | 612 (29.0%) | 633 (28.9%) |  |
| >=2 | 1060 (50.2%) | 1088 (49.6%) |  |
| Gait speed | 0.59 [0.46;0.74] | 0.59 [0.45;0.73] | 0.515 |
| BMI: |  |  | **<0.001*** |
| Normal | 1160 (55.0%) | 1140 (52.0%) |  |
| Underweight | 293 (13.9%) | 201 (9.17%) |  |
| Overweight | 488 (23.1%) | 613 (28.0%) |  |
| obese | 170 (8.05%) | 238 (10.9%) |  |
| Depressive symptoms: |  |  | 0.137 |
| Normal | 1185 (56.1%) | 1180 (53.8%) |  |
| depression | 926 (43.9%) | 1012 (46.2%) |  |
| Cognitive function | 9.00 [5.00;12.5] | 9.00 [5.50;12.5] | 0.186 |
| SPPB | 8.00 [6.00;10.0] | 8.00 [6.00;10.0] | 0.078 |

*Note. ADLs = activities of daily living, BMI= body mass index, SPPB= Short Physical Performance Battery;*

********p < 0.05*
