# Supplementary material for: Novel physical performance-based models for activities of daily living disability prediction among Chinese older community population: a nationally representative survey in China
Source: BMC Geriatr. 2022 Mar 31;22:267. doi: 10.1186/s12877-022-02905-y (PMC8974010; doi:10.1186/s12877-022-02905-y)
Supplement: Supplementary file 2 — Additional file 2: Table S2. The baseline characteristics of participants with complete outcome vs. missing outcome at follow-up. [file 12877_2022_2905_MOESM2_ESM.doc]

**Table A.2** The baseline characteristics of participants with complete outcome vs. missing outcome at follow-up

| Variables | 4-year follow-up | | |
| --- | --- | --- | --- |
|  | Missing data | Full data | *P* value |
| Overall | *N=2111* | *N=2192* |  |
| Gender: |  |  | **<0.001*** |
| Male | 1062 (50.3%) | 892 (40.7%) |  |
| Female | 1049 (49.7%) | 1300 (59.3%) |  |
| Age: |  |  | **0.002*** |
| 60-64 | 740 (35.1%) | 809 (36.9%) |  |
| 65-69 | 497 (23.5%) | 569 (26.0%) |  |
| 70-74 | 401 (19.0%) | 423 (19.3%) |  |
| >=75 | 473 (22.4%) | 391 (17.8%) |  |
| Hukou: |  |  | **0.007*** |
| Agricultural | 1666 (78.9%) | 1802 (82.2%) |  |
| Non-Agricultural | 445 (21.1%) | 390 (17.8%) |  |
| Education: |  |  | 0.151 |
| Illiterate | 814 (38.6%) | 911 (41.6%) |  |
| Primary school | 975 (46.2%) | 973 (44.4%) |  |
| Middle school | 213 (10.1%) | 214 (9.76%) |  |
| High school | 72 (3.41%) | 70 (3.19%) |  |
| College and above | 37 (1.75%) | 24 (1.09%) |  |
| Marital status: |  |  | 0.830 |
| Married/Cohabitated | 605 (76.1%) | 1680 (76.6%) |  |
| widowed | 176 (22.1%) | 468 (21.4%) |  |
| Other | 14 (1.76%) | 44 (2.01%) |  |
| Social activity: |  |  | 0.689 |
| Never | 1151 (54.5%) | 1163 (53.1%) |  |
| Not regularly | 251 (11.9%) | 261 (11.9%) |  |
| Almost Weekly | 206 (9.76%) | 212 (9.67%) |  |
| Almost daily | 503 (23.8%) | 556 (25.4%) |  |
| Self-report health: |  |  | 0.925 |
| Good | 189 (8.95%) | 200 (9.12%) |  |
| Fair | 601 (28.5%) | 616 (28.1%) |  |
| Poor | 857 (40.6%) | 877 (40.0%) |  |
| Very poor | 464 (22.0%) | 499 (22.8%) |  |
| Smoking: |  |  | **0.003*** |
| Never | 1314 (62.2%) | 1482 (67.6%) |  |
| Quit | 311 (14.7%) | 268 (12.2%) |  |
| Less than 20 /day | 233 (11.0%) | 212 (9.67%) |  |
| More than 20 /day | 253 (12.0%) | 230 (10.5%) |  |
| Drinking: |  |  | **<0.001*** |
| Never | 1214 (57.5%) | 1422 (64.9%) |  |
| Quit | 255 (12.1%) | 237 (10.8%) |  |
| Less than once/month | 147 (6.96%) | 116 (5.29%) |  |
| More than once/month | 495 (23.4%) | 417 (19.0%) |  |
| Night sleep: |  |  | 0.447 |
| 6-9 | 1140 (54.0%) | 1216 (55.5%) |  |
| <6 | 789 (37.4%) | 807 (36.8%) |  |
| >=9 | 182 (8.62%) | 169 (7.71%) |  |
| Comorbidity: |  |  | 0.852 |
| 0 | 439 (20.8%) | 471 (21.5%) |  |
| 1 | 612 (29.0%) | 633 (28.9%) |  |
| >=2 | 1060 (50.2%) | 1088 (49.6%) |  |
| Gait speed | 0.59 [0.46;0.74] | 0.59 [0.45;0.73] | 0.515 |
| BMI: |  |  | **<0.001*** |
| Normal | 1160 (55.0%) | 1140 (52.0%) |  |
| Underweight | 293 (13.9%) | 201 (9.17%) |  |
| Overweight | 488 (23.1%) | 613 (28.0%) |  |
| obese | 170 (8.05%) | 238 (10.9%) |  |
| Depressive symptoms: |  |  | 0.137 |
| Normal | 1185 (56.1%) | 1180 (53.8%) |  |
| depression | 926 (43.9%) | 1012 (46.2%) |  |
| Cognitive function | 9.00 [5.00;12.5] | 9.00 [5.50;12.5] | 0.186 |
| SPPB | 8.00 [6.00;10.0] | 8.00 [6.00;10.0] | 0.078 |

*Note. ADLs = activities of daily living, BMI= body mass index, SPPB= Short Physical Performance Battery;*

********p < 0.05*
