# Supplementary material for: Novel physical performance-based models for activities of daily living disability prediction among Chinese older community population: a nationally representative survey in China
Source: BMC Geriatr. 2022 Mar 31;22:267. doi: 10.1186/s12877-022-02905-y (PMC8974010; doi:10.1186/s12877-022-02905-y)
Supplement: Supplementary file 3 — Additional file 3: Table S3. Prediction models of ADL established by logistic regression analysis. [file 12877_2022_2905_MOESM3_ESM.docx]

**Table A.3** Prediction models of ADL established by logistic regression analysis

|  | **Model 1**  **OR（95%*CI）*** | **Model 2**  **OR（95%CI）** | **Model 3**  **OR（95%CI）** | **Model 4**  **OR（95%CI）** | **Model 5**  **OR（95%CI）** | **Model 6**  **OR（95%CI）** |
| --- | --- | --- | --- | --- | --- | --- |
| **Gender** |  |  |  |  |  |  |
| Male | 1.000 (Reference) | 1.000 (Reference) | 1.000 (Reference) | 1.000 (Reference) | 1.000 (Reference) | 1.000 (Reference) |
| female | 1.104(0.796-1.540) | 0.872(0.611-1.250) | 1.004(0.720-1.406) | 1.073(0.772-1.498) | 0.912(0.636-1.312) | 0.902(0.631-1.298) |
| **Age (years)** |  |  |  |  |  |  |
| 60 ~ | 1.000 (Reference) | 1.000 (Reference) | 1.000 (Reference) | 1.000 (Reference) | 1.000 (Reference) | 1.000 (Reference) |
| 65 ~ | 1.084(0.758-1.545) | 1.032(0.720-1.474) | 0.983(0.682-1.409) | 1.025(0.714-1.465) | 0.966(0.670-1.386) | 0.997(0.694-1.427) |
| 70 ~ | 1.874(1.315-2.669)^**^ | 1.749(1.222-2.500)^*^ | 1.613(1.122-2.315)^*^ | 1.701(1.188-2.433)^*^ | 1.573(1.092-2.263)^*^ | 1.629(1.134-2.336)^*^ |
| 75 ~ | 2.636(1.841-3.785)^**^ | 2.331(1.612-3.377)^**^ | 1.753(1.201-3.563)^*^ | 2.161(1.494-3.131)^**^ | 1.693(1.154-2.483)^*^ | 2.010(1.381-2.929)^**^ |
| **BMI** |  |  |  |  |  |  |
| Normal | 1.000 (Reference) | 1.000 (Reference) | 1.000 (Reference) | 1.000 (Reference) | 1.000 (Reference) | 1.000 (Reference) |
| Underweight | 1.173(0.759-1.774) | 1.133(0.732-1.716) | 1.231(0.791-1.874) | 1.153(0.744-1.747) | 1.212(0.779-1.846) | 1.129(0.728-1.711) |
| Overweight | 1.240(0.905-1.691) | 1.281(0.933-1.750) | 1.216(0.883-1.667) | 1.272(0.926-1.739) | 1.232(0.893-1.690) | 1.305(0.949-1.787) |
| Obese | 3.190(2.205-4.589)^**^ | 3.345(2.305-4.829)^**^ | 3.009(2.064-4.365)^**^ | 3.124(2.152-4.511)^**^ | 3.077(2.106-4.473)^**^ | 3.243(2.229-4.697)^**^ |
| **Smoking** |  |  |  |  |  |  |
| Never | 1.000 (Reference) | 1.000 (Reference) | 1.000 (Reference) | 1.000 (Reference) | 1.000 (Reference) | 1.000 (Reference) |
| Quit | 1.194(0.774-1.815) | 1.250(0.810-1.902) | 1.286(0.827-1.972) | 1.256(0.810-1.920) | 1.307(0.840-2.004) | 1.290(0.832-1.971) |
| Less than 20 /day | 1.619(1.040-2.483)^*^ | 1.635(1.049-2.511)^*^ | 1.692(1.080-2.613)^*^ | 1.665(1.066-2.562)^*^ | 1.694(1.081-2.616)^*^ | 1.670(1.069-2.571)^*^ |
| More than 20 /day | 0.875(0.491-1.494) | 0.938(0.526-1.606) | 0.974(0.542-1.679) | 0.942(0.527-1.613) | 0.993(0.552-1.715) | 0.979(0.548-1.680) |
| **Self-report health** |  |  |  |  |  |  |
| Good | 1.000 (Reference) | 1.000 (Reference) | 1.000 (Reference) | 1.000 (Reference) | 1.000 (Reference) | 1.000 (Reference) |
| Fair | 1.126(0.678-1.932) | 1.124(0.675-1.932) | 1.112(0.663-1.925) | 1.056(0.633-1.819) | 1.109(0.661-1.920) | 1.060(0.635-1.828) |
| Poor | 1.257(0.774-2.118) | 1.231(0.756-2.079) | 1.161(0.708-1.972) | 1.158(0.710-1.958) | 1.151(0.702-1.956) | 1.148(0.703-1.943) |
| Very poor | 1.961(1.185-3.357)^*^ | 1.883(1.135-3.230)^*^ | 1.673(1.000-2.892) | 1.768(1.063-3.038)^*^ | 1.652(0.987-2.856) | 1.731(1.040-2.980)^*^ |
| **Depression symptoms** |  |  |  |  |  |  |
| Normal | 1.000 (Reference) | 1.000 (Reference) | 1.000 (Reference) | 1.000 (Reference) | 1.000 (Reference) | 1.000 (Reference) |
| Depression | 1.549(1.192-2.017)^*^ | 1.529(1.176-1.993)^*^ | 1.503(1.151-1.966)^*^ | 1.547(1.189-2.018)^*^ | 1.497(1.147-1.959)^*^ | 1.538(1.181-2.008)^*^ |
| **Cognitive function** | 0.948(0.917-0.979)^*^ | 0.956(0.925-0.988)^*^ | 0.965(0.933-0.998)^*^ | 0.957(0.926-0.989)^*^ | 0.968(0.936-1.002) | 0.962(0.930-0.995)^*^ |
| **Handgrip strength(kg)** | NA | 0.971(0.953-0.988)^*^ | NA | NA | 0.987(0.969-1.006) | 0.979(0.961-0.997)^*^ |
| **SPPB score** | NA | NA | 0.802(0.758-0.847)^**^ | NA | 0.809(0.764-0.856)^**^ | NA |
| **Gait speed (m/s)** | NA | NA | NA | 0.177(0.090-0.340)^**^ | NA | 0.205(0.103-0.395)^**^ |

*Note. y living, evaluated by the Katz ADL scale, BMI= body mass index, CI= confidence interval, SPPB= Short Physical Performance Battery*

*Model 1 incorporated seven predictors, including gender, age, smoking, self-report health condition, BMI, depressive symptoms, and cognitive function. Besides, five physical performance- based models were established based on Model 1, adding handgrip strength (Model 2), SPPB (Model 3), gait speed (Model 4), handgrip strength plus SPPB (Model 5), and handgrip strength plus gait speed (Model 6), respectively.*

*********p < 0.01****；*****p < 0.05*
