# Supplementary figures and images for: Novel physical performance-based models for activities of daily living disability prediction among Chinese older community population: a nationally representative survey in China
Source: BMC Geriatr. 2022 Mar 31;22:267. doi: 10.1186/s12877-022-02905-y (PMC8974010; doi:10.1186/s12877-022-02905-y)

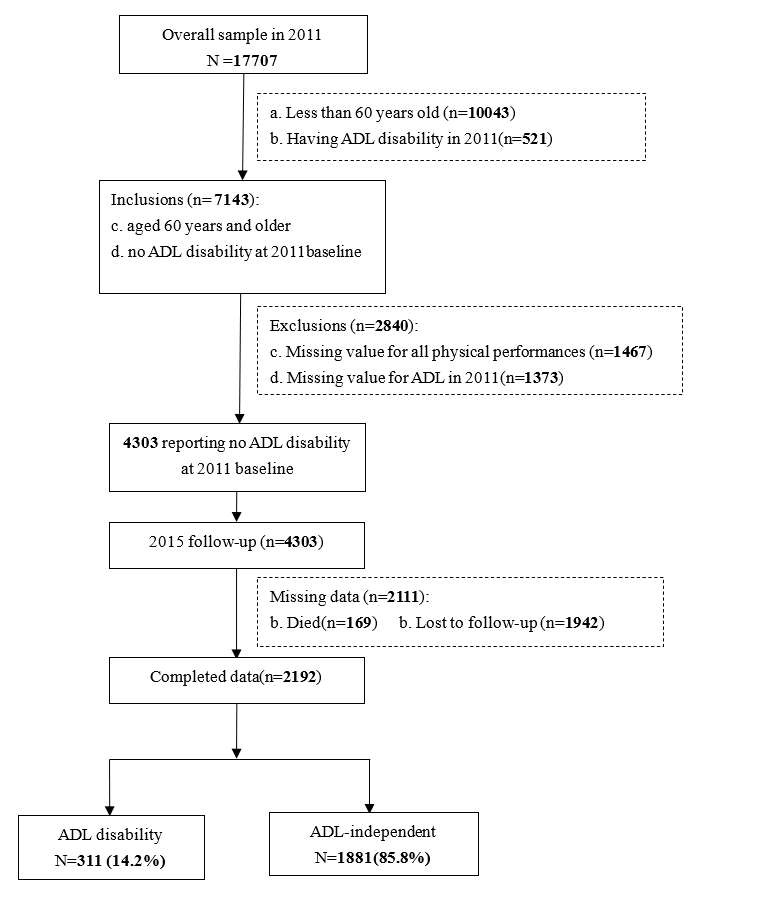

Supplement: Supplementary file 4 — Additional file 4: Figure S1.Study flow. [file 12877_2022_2905_MOESM4_ESM.tiff]

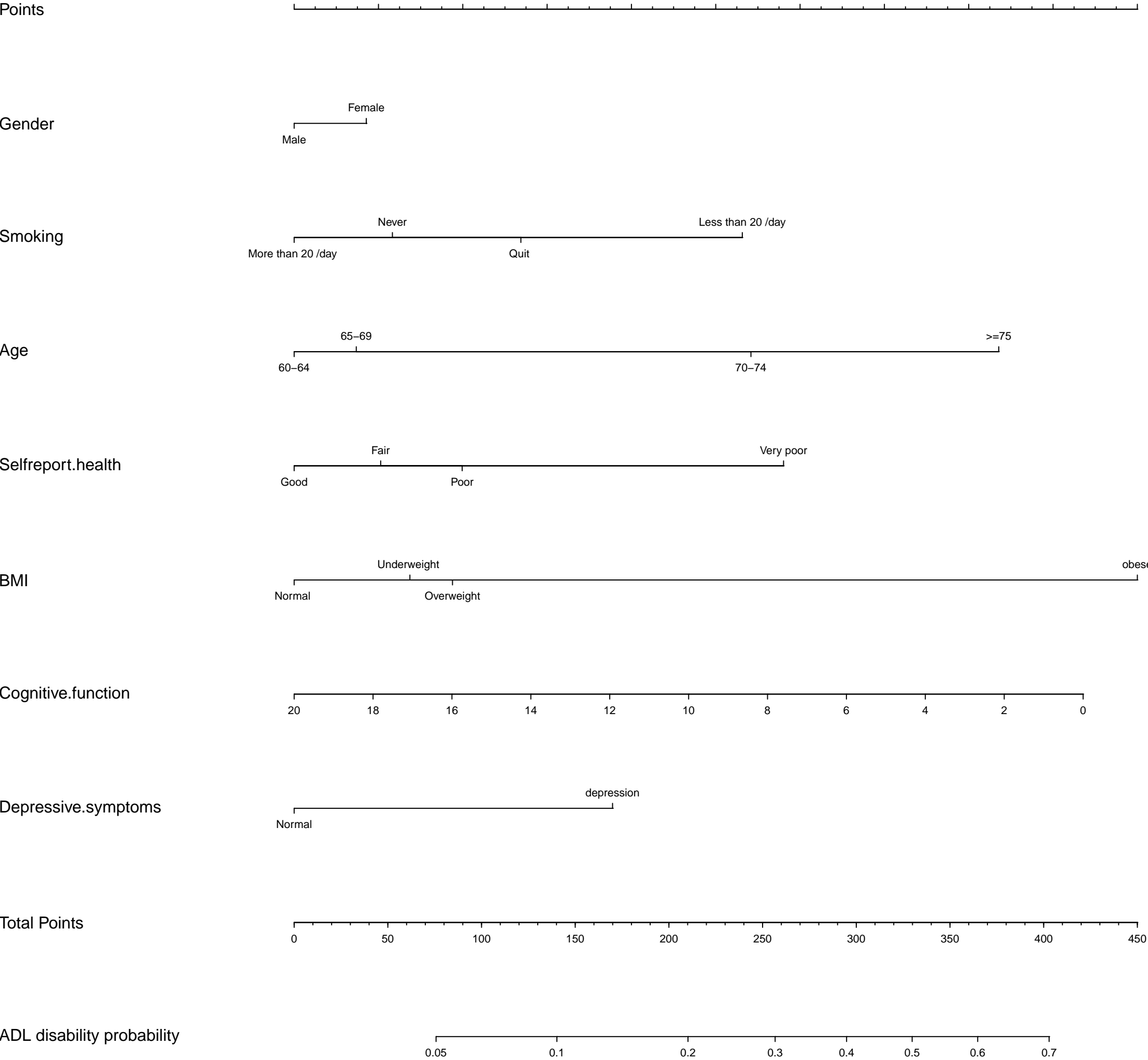

Supplement: Supplementary file 5 — Additional file 5: Figure S2. Nomogram for Model 1(fundamental model). [file 12877_2022_2905_MOESM5_ESM.pdf]

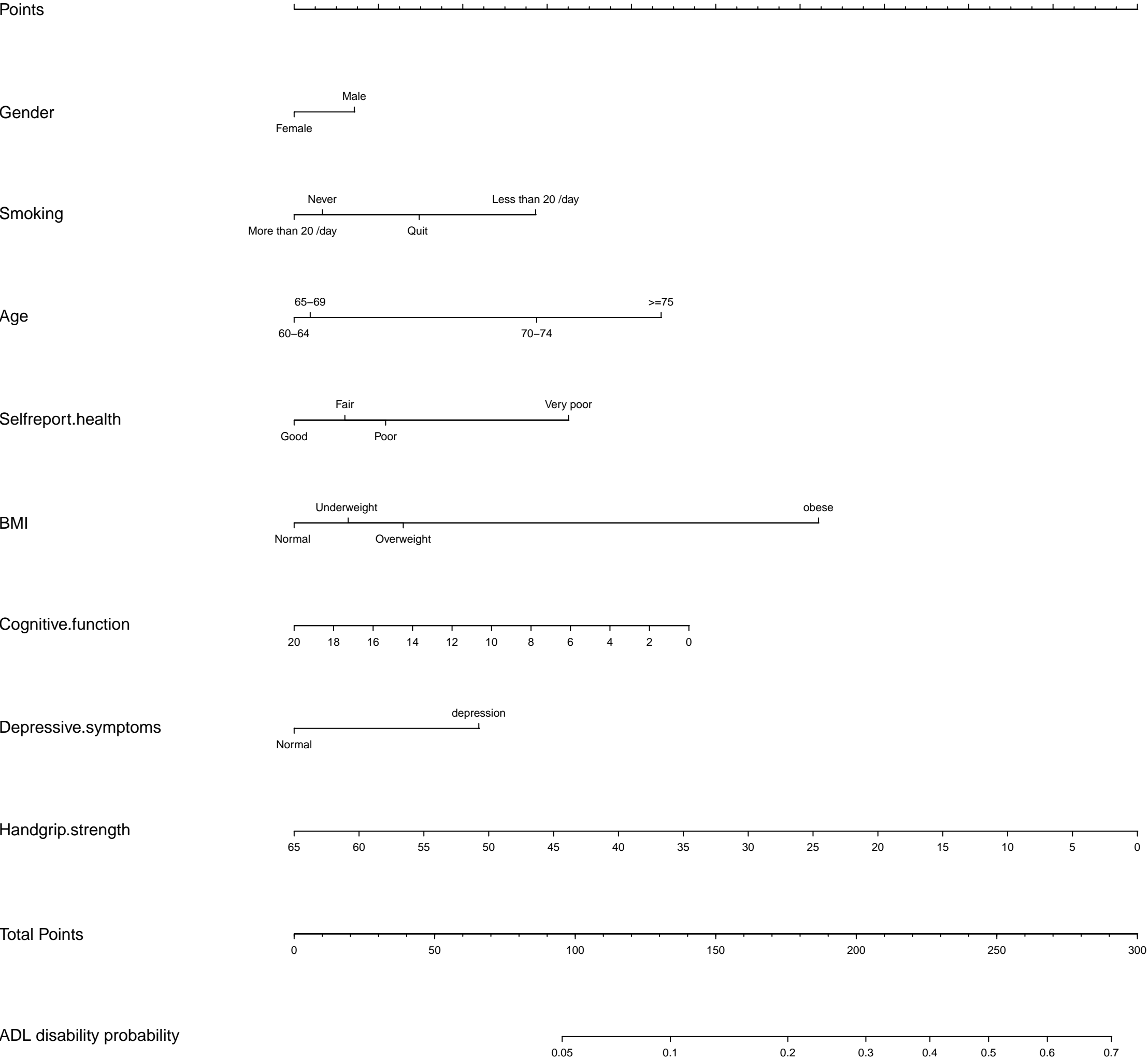

Supplement: Supplementary file 6 — Additional file 6: Figure S3. Nomogram for Model 2 (handgrip strength model). [file 12877_2022_2905_MOESM6_ESM.pdf]

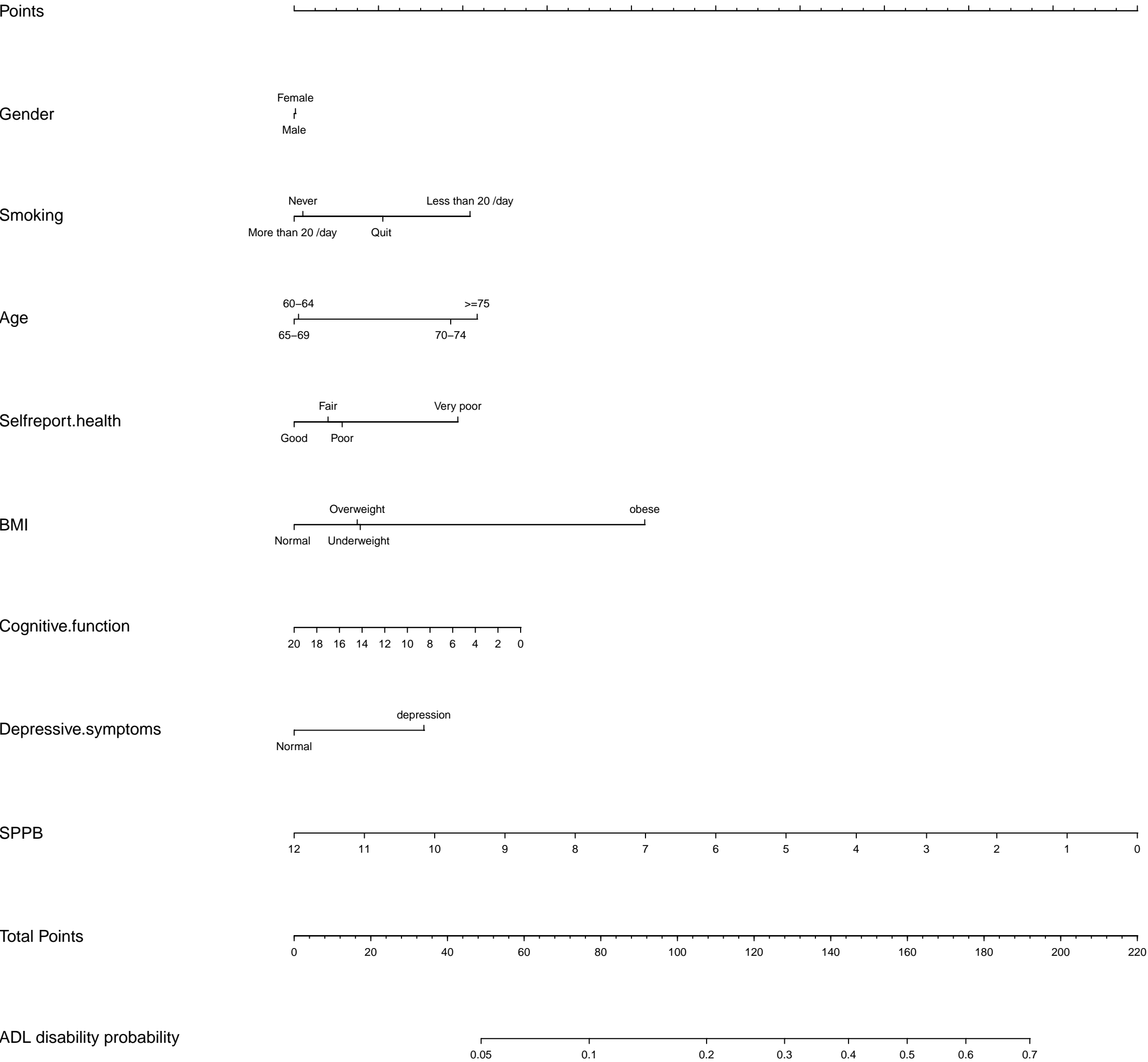

Supplement: Supplementary file 7 — Additional file 7: Figure S4. Nomogram for Model 3(SPPB model). [file 12877_2022_2905_MOESM7_ESM.pdf]

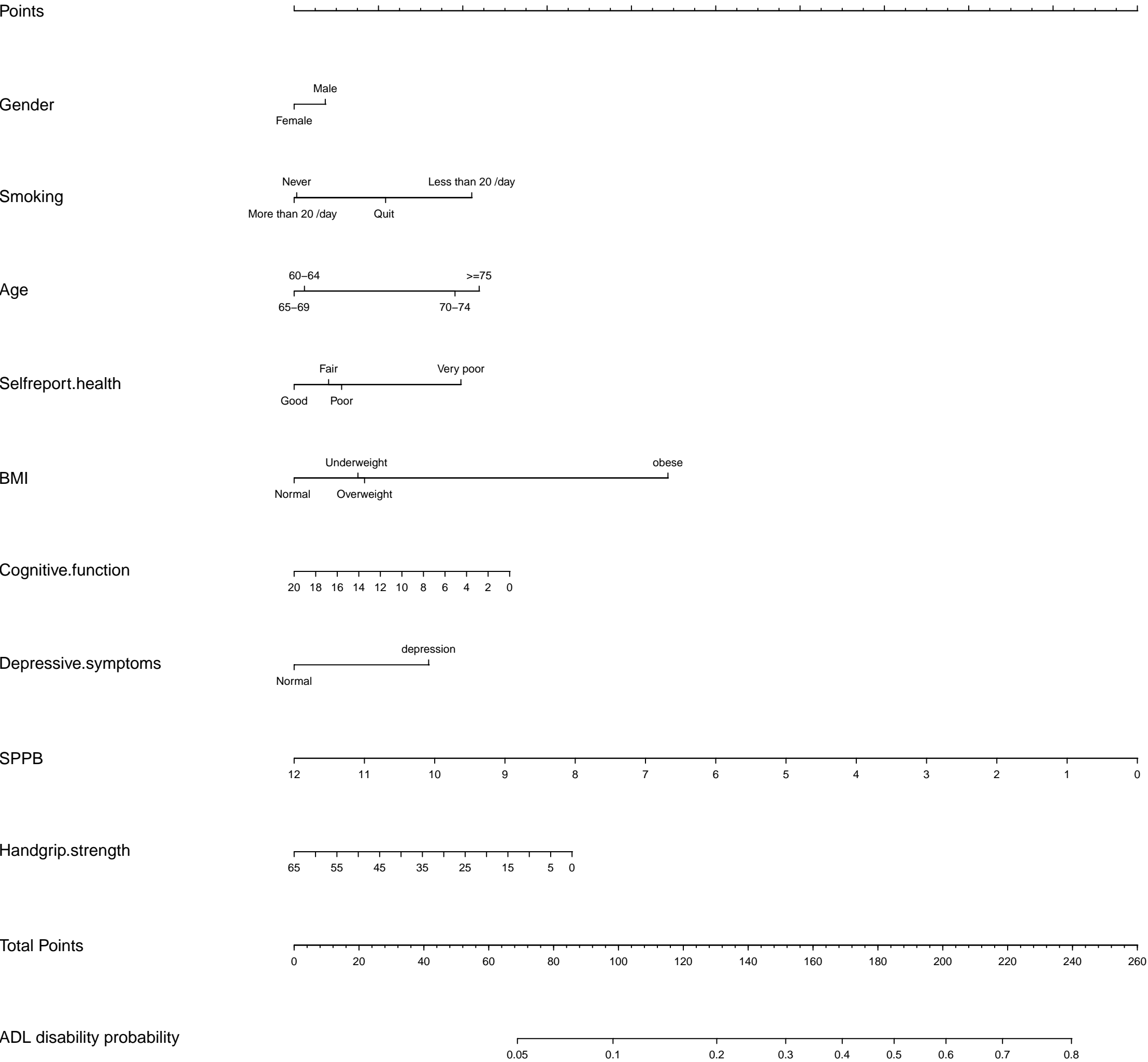

Supplement: Supplementary file 9 — Additional file 9: Figure S6. Nomogram for Model 5 (handgrip strength + SPPB model). [file 12877_2022_2905_MOESM9_ESM.pdf]

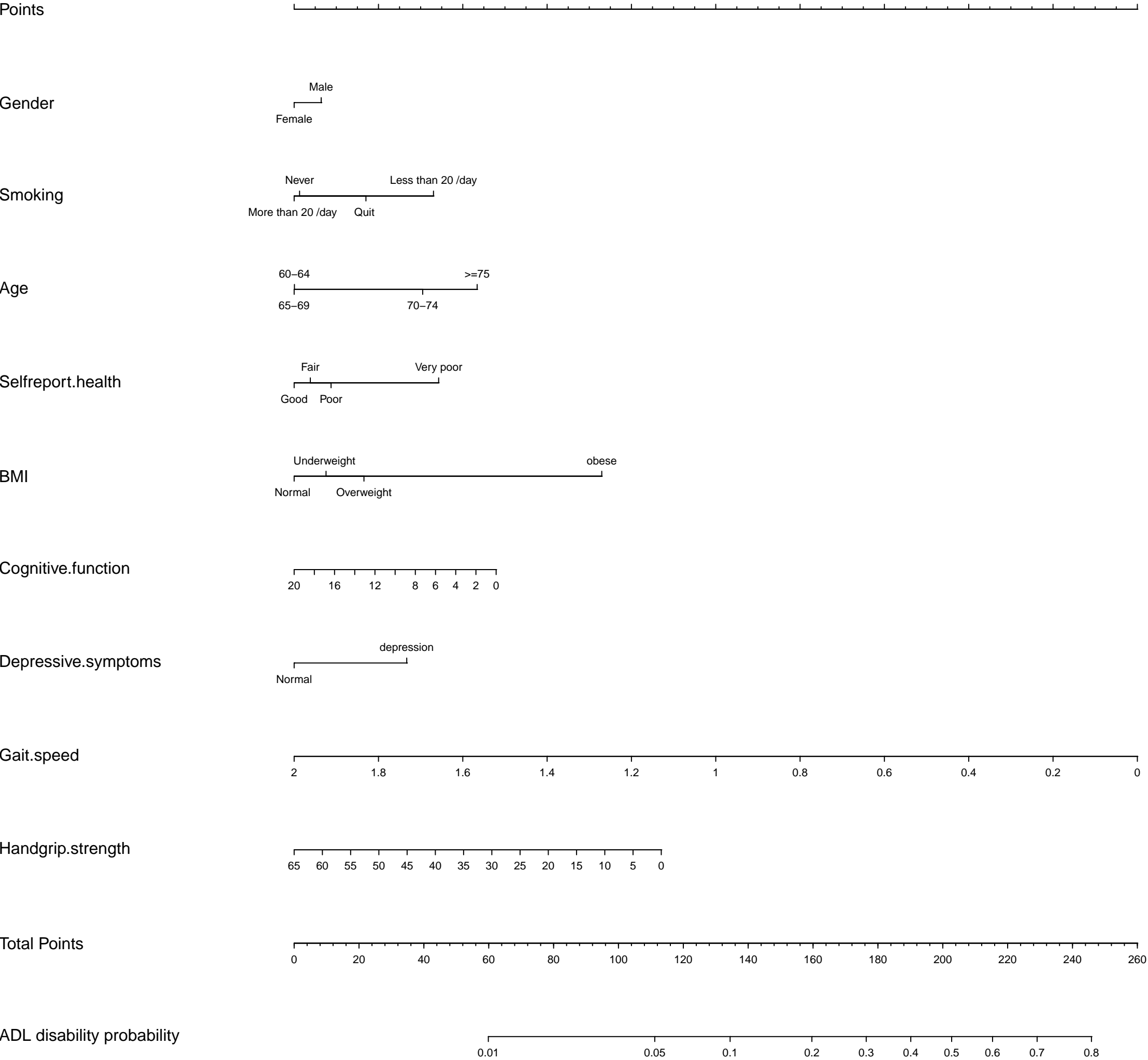

Supplement: Supplementary file 10 — Additional file 10: Figure S7. Nomogram for Model 6(handgrip strength + gait speed model). [file 12877_2022_2905_MOESM10_ESM.pdf]

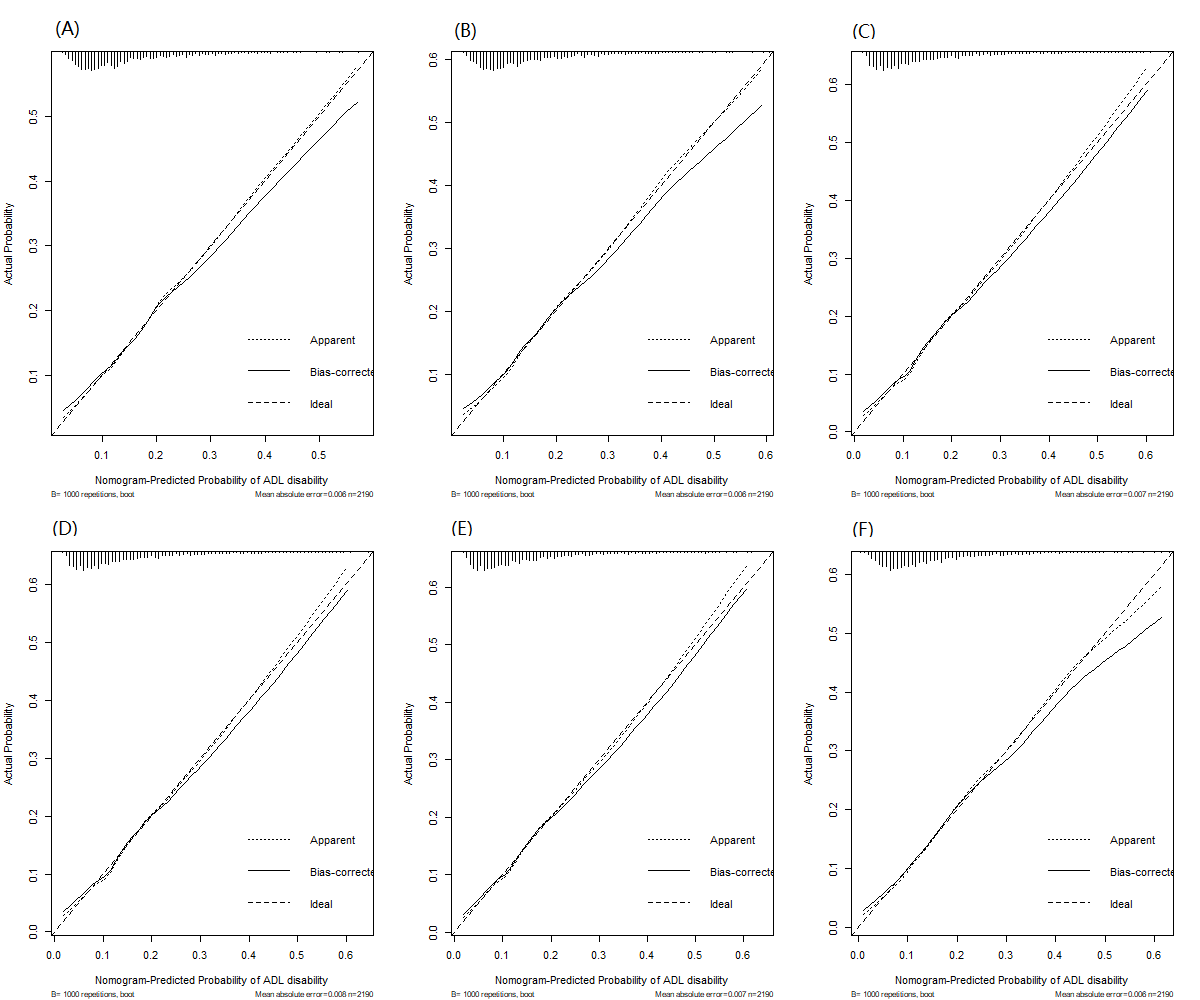

Supplement: Supplementary file 11 — Additional file 11: Figure S8. Bootstrap corrected calibration curves. [file 12877_2022_2905_MOESM11_ESM.tiff]
