# Supplementary material for: Novel physical performance-based models for activities of daily living disability prediction among Chinese older community population: a nationally representative survey in China
Source: BMC Geriatr. 2022 Mar 31;22:267. doi: 10.1186/s12877-022-02905-y (PMC8974010; doi:10.1186/s12877-022-02905-y)
Supplement: Supplementary file 8 — Additional file 8: Figure S5. Nomogram for Model 4(gait speed model). [file 12877_2022_2905_MOESM8_ESM.pdf]

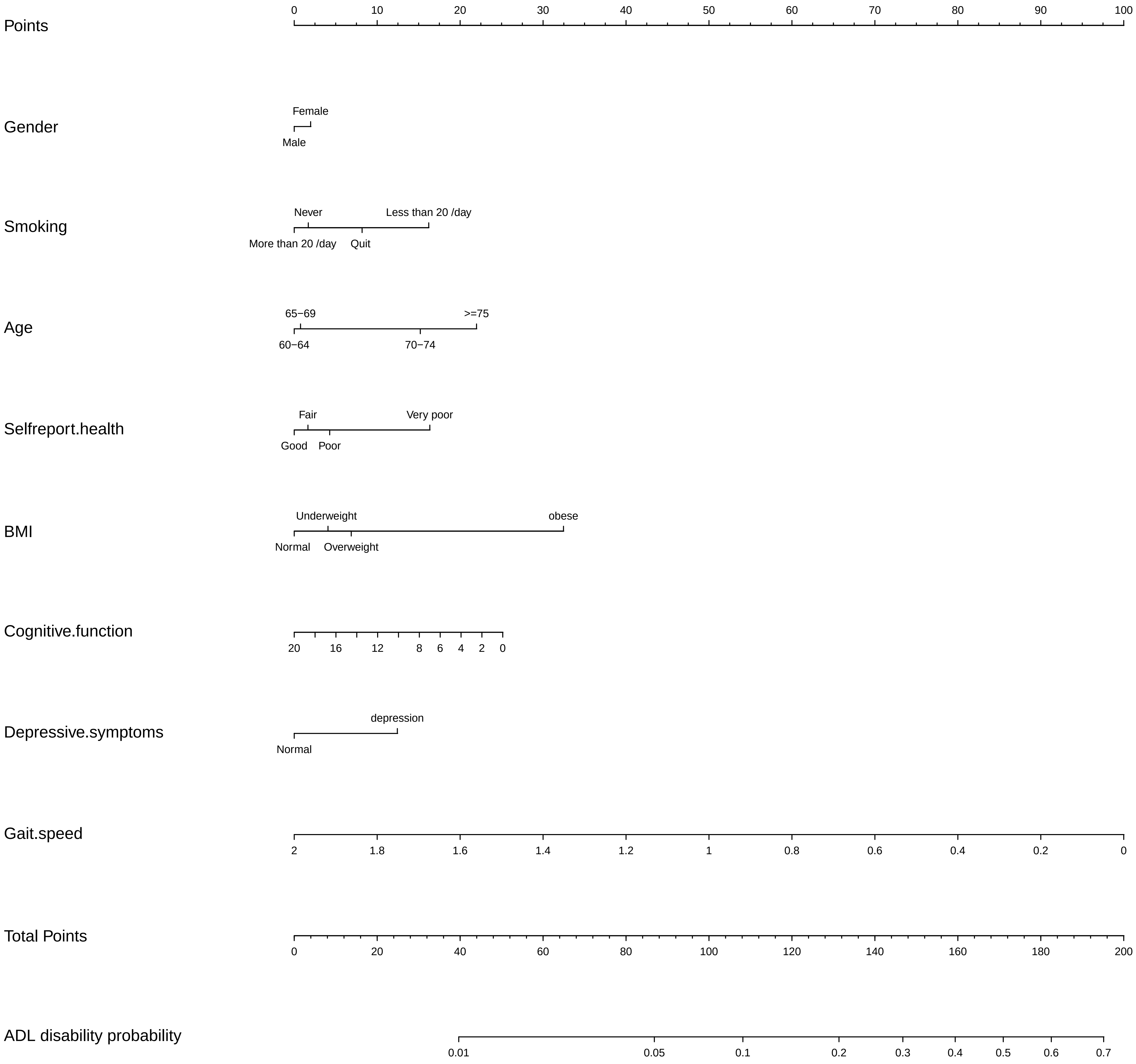
Points
0 10 20 30 40 50 60 70 80 90 100
Gender
Female
Male
Smoking
Never
Less than 20 /day
More than 20 /day
Quit
Age
65−69
>=75
60−64
70−74
Selfreport.health
Fair
Very poor
Good
Poor
BMI
Underweight
obese
Normal
Overweight
Cognitive.function
20 16 12 8 6 4 2 0
Depressive.symptoms
depression
Normal
Gait.speed
2 1.8 1.6 1.4 1.2 1 0.8 0.6 0.4 0.2 0
Total Points
0 20 40 60 80 100 120 140 160 180 200
ADL disability probability
0.01 0.05 0.1 0.2 0.3 0.4 0.5 0.6 0.7
